# Machine learning-enhanced fluorescence signal processing of carbon quantum dots for high-accuracy chemical sensing

**DOI:** 10.1039/d6ra06190g

**Published:** 2026-08-11

**Authors:** Biju Theruvil Sayed, Maharshikumar B. Shukla, Sumit Sharma, Zyad Shaaban, Divya Singhal, Ozodbek Nematov, Ibrokhim Sapaev, Tawfeeq Alghazali, Aseel Smerat, Sahar Bayatinia

**Affiliations:** a Department of Computer Science, Dhofar University PO Box 2509, PCode 211 Salalah Oman; b Department of Chemistry, Faculty of Science, Gokul Global University Sidhpur Gujarat India; c Department of Computer Science and Engineering, Chandigarh University Mohali Punjab India; d Department of Computer Science, University College of Duba, University of Tabuk Duba 71911 Saudi Arabia; e Centre for Research Impact & Outcome, Chitkara University Institute of Engineering and Technology, Chitkara University Rajpura 140401 Punjab India; f Sharda School of Bio-Science & Technology, Sharda University Greater Noida India; g Jizzakh state pedagogical university Jizzakh Uzbekistan; h Department of Physics and Chemistry, Tashkent Institute of Irrigation and Agricultural Mechanization Engineers, National Research University Tashkent Uzbekistan; i Department of Physics and Chemistry, “Tashkent Institute of Irrigation and Agricultural Mechanization Engineers” National Research University Tashkent Uzbekistan; j Chemical Deparment, University of Tashkent for Applied Science Tashkent Uzbekistan; k School of Engineering, Central Asian University Tashkent 111221 Uzbekistan; l The Islamic University in Najaf Najaf Iraq; m Hourani Center for Applied Scientific Research, Al-Ahliyya Amman University Amman 19328 Jordan; n Young Researchers and Elite Club, Tehran Branch, Islamic Azad University Tehran Iran saharbayatinia.academic@gmail.com

## Abstract

Carbon quantum dots (CQDs) exhibit rich photophysical behaviors, including excitation-dependent emission, surface-state variability, and multimodal fluorescence pathways, which complicate accurate, signal interpretation in chemical sensing. Recent advances in machine learning (ML) offer powerful solutions for modeling these complexities and enhancing fluorescence-based detection performance. This review provides a comprehensive analysis of ML-driven methodologies for denoising, spectral decomposition, feature extraction, and high-accuracy classification in CQD fluorescence systems. Mathematical foundations of key ML paradigms are outlined to establish a rigorous framework for signal reconstruction and generalization. Evaluations of recent applications demonstrate how ML enables ultra-low-level analyte detection, interpretable photophysical modeling, and real-time intelligent sensing across chemical and biological environments. Emerging trends—including physics-informed learning, generative data augmentation, autonomous closed-loop sensing, and distributed multimodal architectures—are examined as frontiers poised to redefine CQD fluorescence analytics. Collectively, the integration of ML with CQD photophysics represents a transformative pathway toward robust, adaptive, and next-generation chemical sensing platforms.

## Abstract 2

Carbon quantum dots (CQDs) display complex photophysical behaviors, including excitation-dependent emission, surface-state variability, and multimodal fluorescence pathways, making accurate signal interpretation in chemical sensing challenging.

## Graphical abstract

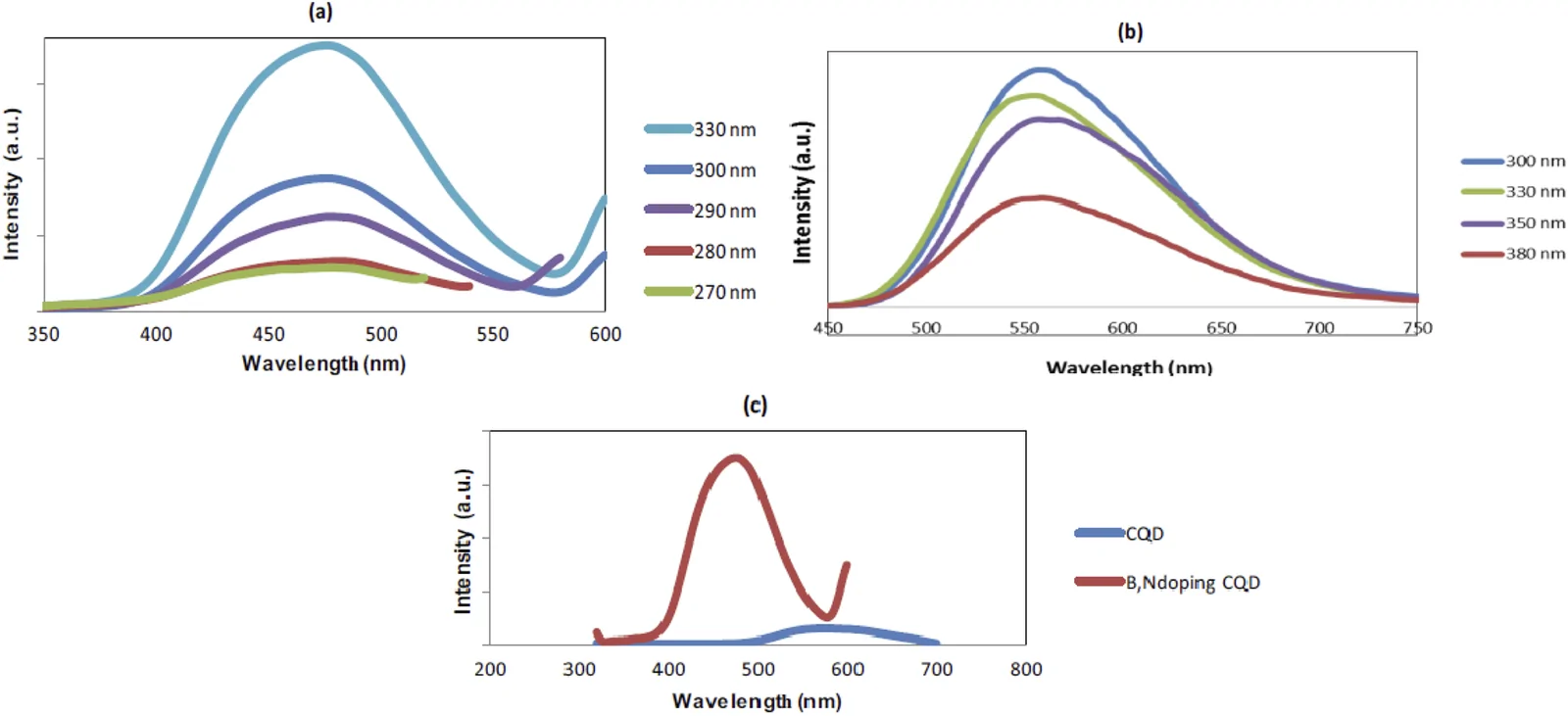

## 1. Introduction

Carbon quantum dots (CQDs) have emerged as one of the most versatile classes of fluorescent nanomaterials due to their low toxicity, chemical robustness, tunable photoluminescence, and compatibility with aqueous and biological environments. Over the past decade, CQDs have been increasingly integrated into chemical sensing platforms, environmental monitoring systems, and biomedical diagnostics.^1–4^ Yet, despite their widespread adoption, CQD-based fluorescence analysis continues to face fundamental limitations stemming from their intrinsic photophysical heterogeneity. Excitation-dependent emission, dynamic surface state distributions, fluctuating quantum yields, and multimodal radiative and non-radiative pathways introduce substantial variability into their spectral signatures.^5–7^ These complexities challenge traditional analytical approaches and hinder the development of robust, high-accuracy sensing technologies capable of reliable performance across diverse chemical environments. As sensing applications push toward ultra-low-level detection and real-time analyte quantification, addressing these photophysical challenges becomes increasingly urgent.^8–10^

Conventional signal processing and chemometric methods—such as linear spectral unmixing, classical filtering, and PCA-based dimensionality reduction—provide only partial solutions. Their inherent assumptions regarding linearity, noise distribution, and stability of emission profiles often fail to capture the nonlinear and environment-dependent behaviors of CQDs.^11–13^ Furthermore, the growing use of multimodal fluorescence readouts, time-resolved spectroscopy, and multiplexed sensing arrays introduces datasets of increasing dimensionality and complexity.^14–16^ These trends expose the limitations of traditional tools, which are neither sufficiently adaptive nor computationally expressive to model intricate fluorescence-analyte interactions. Consequently, there is a rising need for more powerful, data-driven frameworks capable of extracting meaningful information from complex CQD emission landscapes.^17,18^

Recent advances in machine learning (ML) offer a transformative opportunity to overcome these barriers. The ability of ML algorithms to model nonlinear relationships, detect hidden patterns, learn from noisy data, and generalize across heterogeneous spectral conditions aligns precisely with the complexities inherent in CQD fluorescence systems. Beyond simple regression or classification, modern ML techniques enable tasks such as denoising at ultra-low signal levels, spectral decomposition of overlapping emission pathways, multifeature extraction from high-dimensional datasets, and real-time decision-making in intelligent sensing devices.^19–22^ These capabilities represent a major leap beyond the constraints of conventional chemometric approaches and open new avenues for enhancing detection accuracy in CQD-based sensing technologies.

Existing reviews have substantially advanced the broader understanding of computational modeling, ML-assisted QD research, and data-driven carbon-dot development. General QD reviews mainly discuss first-principles calculations, mathematical modeling, and AI-based approaches for predicting or controlling nanostructure properties.^23^ CQD-oriented reviews have further emphasized ML-assisted synthesis optimization, water-related applications, electrochemistry, and predictive modeling of precursor–property–performance relationships.^24,25^ In addition, carbon-dot-focused ML reviews provide useful perspectives on data-driven synthesis and application optimization across broader CD systems, although they are not specifically centered on CQD fluorescence signal processing.^27^ Similarly, broader ML-QD surveys have mapped applications across device tuning, high-throughput characterization, sensing, photonics, and reduced-order modeling, while also highlighting dataset bias, limited transferability, and insufficient benchmarking.^26^ Compared with these contributions, the present review has a narrower material and application scope, but a more focused analytical objective: connecting CQD photophysical complexity with ML-enabled fluorescence signal processing.

This distinction is important because excitation-dependent emission, spectral broadening, multimodal radiative–nonradiative coupling, environmental sensitivity, and temporal drift directly limit the reliability of CQD fluorescence sensing. Existing reviews provide valuable background on QD computation, CQD informatics, and CD-oriented ML strategies, but they rarely formalize how these intrinsic optical instabilities should be translated into ML model design, validation, interpretability, uncertainty estimation, and cross-domain generalization.^23–27^ Therefore, this review contributes a complementary perspective by linking material-level emission heterogeneity with algorithmic signal recovery and sensing performance. Rather than positioning ML as a substitute for photophysical understanding, it frames ML as a compensatory and interpretive layer that can extract weak, overlapping, and noisy fluorescence information when supported by physically informed constraints, standardized datasets, and rigorous validation. This integrated viewpoint may help guide the development of more adaptive, interpretable, and transferable CQD-based fluorescence sensing platforms.

## 2. Photophysical complexity of CQDs: challenges in spectral variability, noise, and multimodal emission dynamics

### 2.1. Intrinsic heterogeneity in electronic structure and its impact on emission behavior

CQDs are inherently heterogeneous nanostructures, displaying wide distributions in size, surface states, oxidation degree, and defect density. Unlike traditional semiconductor quantum dots with well-defined crystal lattices and discrete energy levels, CQDs exhibit a hybrid electronic landscape comprising sp^2^–sp^3^ carbon domains, localized defect sites, and oxygen/nitrogen functionalities.^28–30^ This structural complexity produces a convoluted manifold of emissive states, often competing or interacting through Förster resonance energy transfer (FRET), phonon-mediated relaxation, and defect-assisted recombination. Consequently, fluorescence emission from CQDs cannot be described by a single-bandgap model but emerges characteristics.^31,32^

This intrinsic heterogeneity produces spectral variability even when CQDs are synthesized under seemingly identical conditions. Slight deviations in precursor composition, pyrolysis temperature, or solvent exposure can significantly alter emissive centers. Moreover, the coexistence of particle-core emission and surface-state emission leads to excitation-dependent fluorescence—a hallmark of CQD photophysics that complicates straightforward interpretation. The broad, overlapping emission bands resulting from these hybrid pathways often mask subtle changes induced by analytes or environmental stimuli.^33–35^

Critically, this variability poses a fundamental challenge for quantitative fluorescence sensing. When each batch of CQDs exhibits distinct emission fingerprints, signal reproducibility becomes difficult to guarantee across experiments or laboratories. Without robust calibration strategies—or advanced computational correction—instrumental variations or material inconsistencies can be misinterpreted as analyte-induced effects. Thus, intrinsic heterogeneity is not merely a synthetic artifact but a core physical limitation that must be thoroughly understood to enable reliable optical sensing.

**Fig. 1:**
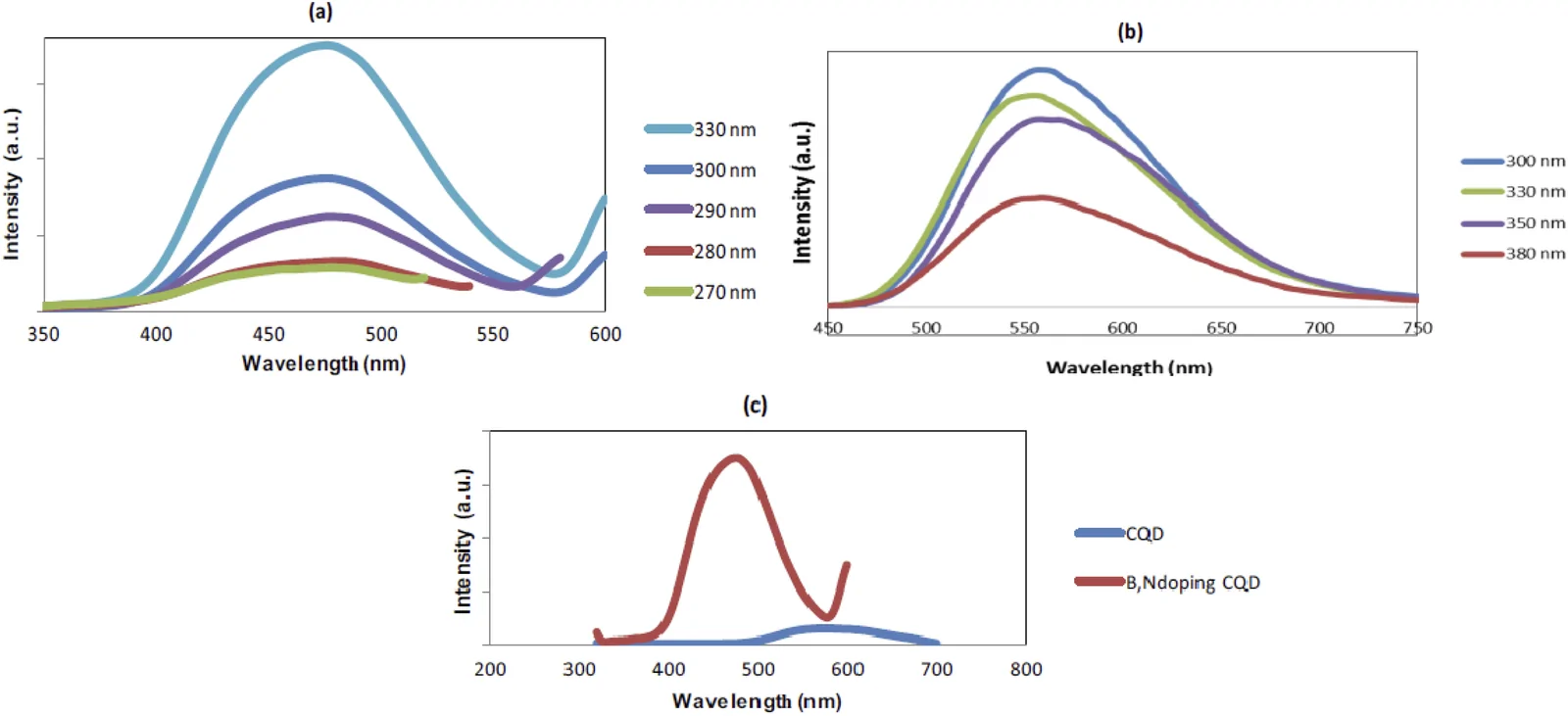
(a) PL spectra of B,N-CQDs at excitation wavelengths 270–330 nm. (b) PL spectra of urea-derived CQDs at 300–380 nm. (c) Comparative emission at 330 nm, highlighting stronger, more stable fluorescence in B,N-CQDs. Adapted with permission from ref. 34. © 2023 Nature.

Fig. 1 collectively illustrates how the heterogeneous electronic structure of the synthesized CQDs governs their excitation-dependent photoluminescence. In panel (a), B,N-CQDs display a consistent emission maximum near 470 nm across excitation wavelengths from 270–330 nm, while the gradual decrease in intensity at shorter excitations reflects changes in population of surface-derived emissive states. Panel (b) shows that urea-derived CQDs exhibit a contrasting trend: their emission becomes stronger at lower excitation wavelengths and shifts toward longer wavelengths, indicating a broader and deeper distribution of trap states associated with size variability and surface defects. Panel (c) directly compares the two materials at 330 nm excitation, revealing a pronounced red-shift to around 600 nm for undoped CQDs, in contrast to the stable blue-green emission of B,N-CQDs. The substantially higher fluorescence intensity of B,N-CQDs highlights the enhanced radiative recombination afforded by heteroatom-induced modulation of electronic domains.

### 2.2. Environmental susceptibility: influence of pH, ionic strength, solvent polarity, and surface adsorption

CQD fluorescence is highly sensitive to environmental parameters, which can significantly modulate emission intensity, peak position, and spectral shape. The surface of CQDs is typically rich in hydroxyl, carboxyl, amino, and carbonyl groups. These moieties interact dynamically with protons, ions, and solvent molecules, altering the local electronic density of states. For instance, protonation/deprotonation equilibria under varying pH conditions can shift surface-state energy levels, resulting in blue-shifts, red-shifts, or quenching. Similarly, ionic strength influences electrostatic screening around CQDs, potentially suppressing non-radiative recombination or, conversely, promoting aggregation-induced quenching.^36–38^

Solvent polarity exerts another layer of modulation. Polar environments may stabilize certain excited states, increasing radiative decay rates, while hydrophobic microenvironments may restrict solvent relaxation, leading to enhanced vibronic features. These effects are often non-linear and difficult to decouple experimentally. Additionally, surface adsorption of biomolecules, metal ions, or organic ligands can introduce new charge-transfer pathways, fundamentally altering emission dynamics. Even trace contaminants—including dissolved oxygen or residual synthesis byproducts—can influence emission lifetimes and intensities.^39,40^

The environmental susceptibility of CQDs complicates their deployment in real-world sensing applications, where matrices such as sweat, urine, or natural water introduce a multitude of uncontrolled variables. This lack of environmental robustness poses a major barrier to standardization and comparative analysis across studies. Furthermore, the same analyte may produce markedly different optical responses depending on microenvironmental context, leading to ambiguous or contradictory results.^41–43^ Therefore, a deep mechanistic understanding of microenvironment-induced perturbations is essential to decouple analyte-specific interactions from background physicochemical effects.

**Fig. 2:**
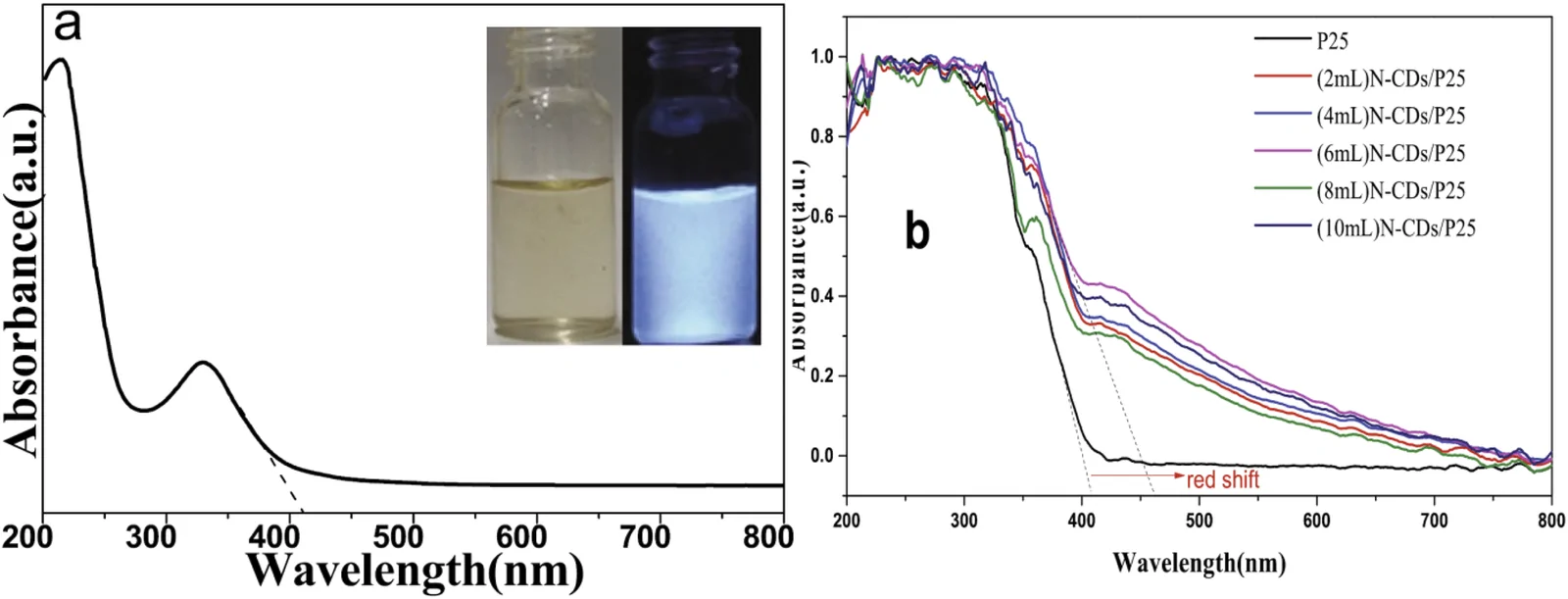
(a) UV-vis absorption of N-CDs and photographs under natural light and 365 nm excitation. (b) UV-vis DRS spectra of P25 and N-CDs/P25 showing enhanced visible absorption and red-shifted edges upon N-CDs deposition. Adapted with permission from ref. 42. © 2019 Nature.

The optical response of the N-CDs shown in Fig. 2 provides direct evidence of their susceptibility to environmental excitation conditions, as the broad *n*–π* absorption tail spanning 280–450 nm enables multiple excitation channels that can selectively populate distinct emissive states under varying irradiation environments. In Fig. 2a, the coexistence of a strong π–π* transition at 228 nm and a wide defect-related absorption band results in pronounced excitation-dependent behavior, reflected in the visible-UV photos where the dispersion appears yellow under natural light but switches to a blue emission under 365 nm illumination. Such color variation illustrates the sensitivity of surface and defect states to the spectral content of the surrounding light field. Similarly, Fig. 2b shows that coupling N-CDs with P25 induces a red-shifted absorption edge and enhanced visible-light harvesting, indicating that interfacial interactions and local optical environments can significantly modulate electronic transitions and light–matter interactions. These observations collectively highlight the environmental dependence of the absorption–excitation landscape in N-CDs systems.

### 2.3. Multimodal emission pathways and interference among radiative and non-radiative processes

The fluorescence behavior of CQDs is governed by a complex interplay among radiative emission, non-radiative relaxation, surface-state recombination, and defect-assisted transitions. Unlike crystalline semiconductor nanocrystals where excitonic recombination dominates, CQDs exhibit multiple emission pathways coexisting across different spatial domains within the same nanoparticle. Core-state emission, typically associated with sp^2^ conjugated domains, competes with surface-state emission arising from oxygenated or nitrogenated defect sites. The coexistence of these pathways generates non-trivial emission landscapes, making spectral interpretation inherently difficult.^44–47^

Moreover, non-radiative decay channels—including phonon coupling, defect trapping, and photoinduced electron transfer—introduce quenching phenomena that are highly sensitive to external stimuli. Environmental quenchers such as dissolved oxygen or electron-accepting analytes can activate these pathways, masking or mimicking analytical signals. Additionally, photon reabsorption and inner-filter effects may distort spectral outputs, particularly in concentrated or strongly absorbing samples.^48,49^ These distortions often produce artifacts such as apparent emission shifts or non-linear calibration curves, which can be misinterpreted as chemical specificity.

A major challenge arises from the interdependence of these processes. Modifying surface chemistry to enhance brightness may inadvertently introduce trap states that enhance non-radiative pathways. Conversely, reducing defect density to increase emission stability may eliminate useful surface-state transitions that are responsible for analyte sensitivity.^50,51^ This trade-off underscores the need for holistic optimization strategies that account for cross-coupling among pathways.

Understanding the competition between emission channels is critical for designing CQD-based sensors with predictable behavior. Without disentangling these pathways, researchers may attribute observed spectral responses solely to analyte interactions, overlooking the contribution of intrinsic photophysical interference mechanisms.^52,53^

**Fig. 3:**
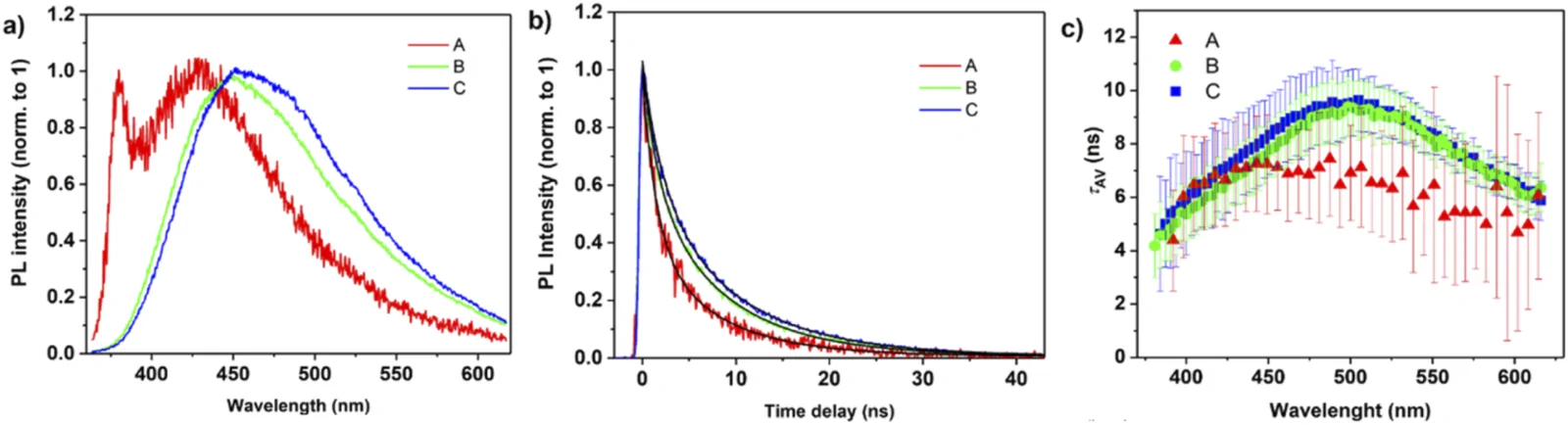
Time-integrated PL spectra (a), biexponential PL decays at emission maxima (b), and wavelength-dependent average lifetimes *τ*_AV_ (c) of samples A–C, revealing multiple radiative and nonradiative pathways and pyrolysis-time-dependent modulation of emissive and defect-mediated states in CQD ensembles. Adapted with permission from ref. 53. © 2025 Elsevier B.V.

Fig. 3 provides detailed insight into the multimodal emission pathways governing the photophysics of the CQD ensembles synthesized at different pyrolysis times. The time-integrated PL spectra of samples A, B, and C (Fig. 3a) exhibit comparable spectral shapes, yet the differences in intensity and slight red shifts reflect variations in the population of emissive *versus* non-emissive states. Time-resolved PL decays recorded at the emission maxima (Fig. 3b) clearly deviate from a single-exponential behavior and require two-exponential fits, indicating the coexistence of multiple radiative and nonradiative recombination channels within the ensembles. Such multi-component decays are characteristic of particle populations exhibiting heterogeneous distributions of defect-related and intrinsic emissive states. The wavelength-dependent average lifetimes *τ*_AV_ (Fig. 3c) further support this interpretation, as their non-monotonic spectral evolution reveals distinct energy-dependent contributions of surface-derived and core-related pathways. Notably, the pronounced differences in *τ*_AV_ among samples A–C correlate with the activation or suppression of competing emissive channels as pyrolysis time modulates defect saturation, structural ordering, and the fraction of dark particles. Overall, these results demonstrate that variations in pyrolysis conditions tune the balance of parallel emission pathways *via* changes in radiative efficiency and defect-mediated recombination.

### 2.4. Temporal instabilities: photobleaching, fluorescence intermittency, and long-term drift

Temporal instability remains one of the most underappreciated yet critical limitations in CQD fluorescence. Photobleaching—typically viewed as a minor issue compared to organic dyes—still occurs in CQDs, especially when surface defects participate in photochemical reactions under prolonged illumination. Oxidative degradation of emissive centers can gradually decrease fluorescence intensity, introducing time-dependent drift in analytical measurements. This is particularly problematic in kinetic assays or portable sensing platforms where temporal consistency is essential.^54–56^

Fluorescence intermittency (blinking), while extensively studied in semiconductor quantum dots, is also observed in CQDs though in more complex and less predictable forms. Blinking in CQDs may arise from stochastic trapping of charge carriers in surface or defect states, transient passivation by environmental species, or fluctuations in local solvent conformation.^57,58^ These intermittencies generate noise in time-resolved fluorescence measurements, degrading signal-to-noise ratios and complicating quantitative analysis of trace analytes.

Long-term spectral drift presents another challenge. Over timescales of hours to days, CQDs may undergo surface reorganization, ligand desorption, or solvent-induced restructuring. These changes subtly shift emission maxima or alter band shapes, making it difficult to maintain calibration stability. Drift effects undermine the reproducibility of sensing outputs and require frequent recalibration—an approach incompatible with large-scale deployment or continuous monitoring.^59,60^

Critically, these temporal instabilities often interact. For example, mild photobleaching may amplify blinking behavior by increasing defect density, or solvent-induced drift may alter susceptibility to quenching. Such couplings introduce systemic noise sources that cannot be eliminated through conventional experimental controls. Therefore, temporal stability must be considered not only as an operational challenge but as an intrinsic photophysical barrier that limits the precision and robustness of CQD-based fluorescence sensing.

CQDs exhibit complex photophysical behavior arising from heterogeneous electronic structures, multimodal emission pathways, and strong environmental sensitivity (Table 1). Their fluorescence is highly responsive to pH, ionic strength, solvent polarity, and surface adsorption, leading to nonlinear and often unpredictable spectral variations. Temporal instabilities—including photobleaching, blinking, and long-term drift—further degrade reproducibility. These intertwined effects generate substantial noise, spectral overlap, and variability, posing fundamental challenges for achieving accurate, stable, and quantitative fluorescence sensing across diverse chemical matrices.

**Table 1:** Key photophysical challenges in CQDs and their implications for fluorescence sensing

Photophysical challenge	Underlying mechanism	Experimental manifestation	Impact on fluorescence sensing	Core limitation created
Size and surface heterogeneity	sp^2^/sp^3^ domains, defect variability	Broad, overlapping PL bands	Reduced spectral specificity	Batch-to-batch inconsistency
Excitation-dependent emission	Multiple emissive centers	Wavelength-dependent PL shifts	Ambiguous analyte responses	Difficult calibration
Surface chemical variability	Protonation/charge transfer	pH-dependent shifts, quenching	Matrix-dependent output	Poor reproducibility
Solvent polarity sensitivity	Solvent relaxation dynamics	Peak shifts, vibronic changes	Instability across media	Limited portability
Ionic strength interactions	Electrostatic screening	Aggregation or quenching	Signal unpredictability	Context-dependent responses
Surface adsorption effects	Ligand/analyte binding	New emission or quenching channels	False-positive signals	Low selectivity
Multimodal emission pathways	Core *vs.* surface states	Overlapping radiative channels	Difficult deconvolution	Mechanistic ambiguity
Non-radiative decay competition	Phonon coupling, traps	Intensity quenching	Reduced SNR	Sensitivity limits
Photobleaching dynamics	Oxidative surface reactions	Gradual intensity loss	Time-dependent drift	Calibration breakdown
Fluorescence intermittency	Stochastic trapping	Blinking noise	Poor temporal stability	Reduced analytical precision
Long-term spectral drift	Ligand loss, surface rearrangement	Peak shifts over hours/days	Unstable sensor output	Poor long-term reliability

## 3. ML paradigms for fluorescence signal reconstruction: mathematical and algorithmic foundations

### 3.1. Limitations of classical modeling and the rise of data-driven methods

Classical fluorescence signal analysis is traditionally built upon parametric formulations that assume each emission feature can be approximated by analytically defined basis functions. The Gaussian mixture formulation:^61–63^1
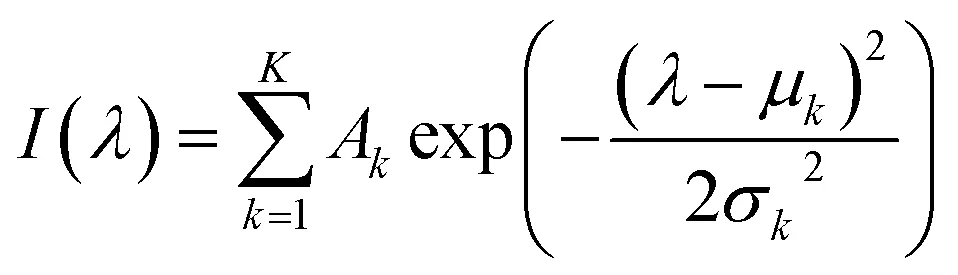


Represents the dominant model used for describing peak-like structures. Although it offers computational simplicity and allows analytical differentiation and integration, the model inherently assumes that spectral features are symmetric, unimodal, and stationary. Real fluorescence spectra, however, often display asymmetric broadening, non-Gaussian tails, and temporally varying structures that violate these assumptions.

Noise modeling constitutes another major limitation. Classical approaches typically assume additive noise:^64–66^

2 *I*_obs_(*λ*) = *I*_true_(*λ*) + *ε*(*λ*)

Which oversimplifies the true behavior of photon statistics, electronic background interference, and detector-dependent distortions. In practice, noise may be multiplicative, heteroscedastic, wavelength-dependent, or originate from nonlinear instrument response curves.

Moreover, traditional fitting and baseline reduction algorithms rely heavily on predefined peak counts, fixed functional forms, and user-selected parameters. This introduces subjectivity and restricts the capacity to reveal latent relationships. Classical models struggle particularly with overlapped peaks, nonlinear interactions between excitation and emission processes, and dense multicomponent spectra.^67,68^

These persistent weaknesses collectively motivated the shift toward machine-learning-based paradigms. ML models make no explicit assumptions about the spectral shape, noise distribution, or linearity of interactions. Instead, they infer structure empirically through optimization, enabling them to capture non-stationary and highly nonlinear patterns. This transition reflects a broader methodological change in signal processing: replacing rigid analytical prescriptions with data-driven representations capable of adapting to complex and irregular spectral landscapes.

### 3.2. Machine learning-based denoising and reconstruction

ML models address the limitations of classical denoising by learning a flexible nonlinear function that maps noisy spectra to their clean counterparts:^69–71^

3 *Î*_clean_ = *f*_*θ*_(*I*_noisy_)

This formulation allows neural networks to approximate highly complex functional relationships, including wavelength-dependent distortions, structured noise, and overlapping spectral signatures. Unlike traditional smoothing filters, which trade resolution for noise reduction, ML models explicitly optimize reconstruction accuracy.

Autoencoders, for instance, learn latent representations that isolate essential spectral structures from noise. Networks are trained by minimizing reconstruction error using a model such as:^72–74^4
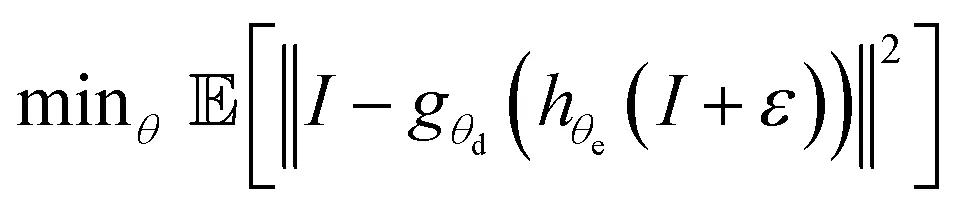
where the encoder compresses the spectrum and the decoder reconstructs it. CNN-based architectures further introduce spatial locality, enabling the model to detect subtle motif variations along the wavelength axis. These convolutional filters effectively capture curvature patterns, inflection points, and fine-scale spectral ripples that classical models typically overlook.

Transformers offer another advantage by modeling long-range dependencies, allowing the reconstruction network to consider relationships between distant wavelengths. This becomes important when spectra contain broad emission envelopes or correlated sub-bands.

Despite these benefits, ML models require diverse training data to avoid overfitting to specific noise characteristics. If environmental or instrumental conditions differ significantly from the training distribution, reconstruction may deteriorate. Therefore, cross-instrument calibration data, augmentation strategies, and physics-informed constraints are essential for robust deployment.^75,76^ Overall, ML-based reconstruction provides a powerful framework, significantly outperforming traditional denoising methods by combining nonlinear modeling capacity with explicit optimization of spectral fidelity.

### 3.3. Feature engineering and dimensionality reduction

Feature engineering transforms raw fluorescence spectra—often consisting of hundreds or thousands of intensity measurements—into compact representations that preserve essential structure. Classical dimensionality-reduction methods rely on linear decompositions. Principal Component Analysis (PCA), for example, expresses a spectrum as:5
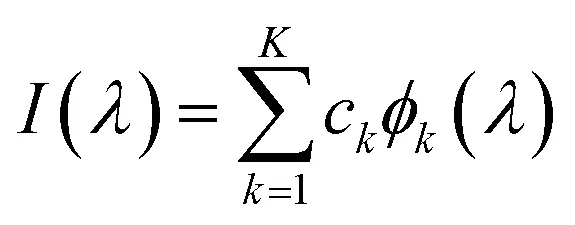
where the principal components *ϕ*_*k*_ capture directions of maximal variance.^77,78^ PCA is computationally efficient and interpretable, but the assumption of linearity restricts its ability to model nonlinear spectral manifolds.

In practice, fluorescence spectra frequently inhabit curved, non-Euclidean manifolds due to nonlinear excitation–emission interactions, instrumental effects, and multi-component blending. Nonlinear embedding algorithms such as Isomap, Laplacian Eigenmaps, and UMAP estimate intrinsic geometry by approximating geodesic distances. These methods preserve local structure and uncover latent patterns that linear projection methods suppress.

Deep learning approaches extend this further. Variational Autoencoders (VAEs) embed spectra into a probabilistic latent space, where the latent variable:6
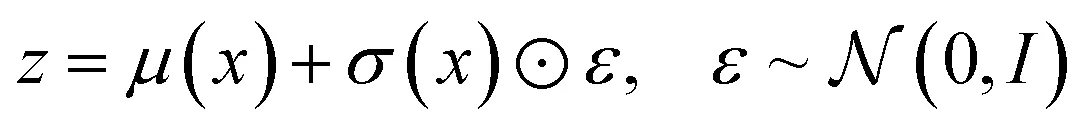


Represents the spectrum in a compressed but structured manner. Such latent spaces often cluster spectra based on subtle variations in peak shape, curvature, or intensity patterns that are challenging to capture *via* manual feature engineering.^79–81^

However, nonlinear embeddings can sacrifice interpretability. Latent variables may not correspond directly to physical spectral attributes, which can complicate downstream analysis. Additionally, embedding stability may decrease when input spectra originate from different instruments, environmental conditions, or acquisition settings. Thus, while feature-learning models significantly enhance representational quality, careful consideration of interpretability, data normalization, and cross-domain robustness is necessary for reliable dimensionality reduction.

### 3.4. Bias, generalization, and physics-informed ML constraints

ML models applied to fluorescence spectra are highly sensitive to biases within the training dataset. When the statistical distribution of training data differs from that of deployment data—often due to instrument variation, environmental changes, or acquisition settings—generalization is compromised. Formally:

7 *p*_train_(*x*) ≠ *p*_test_(*x*) ⇒ *f*_*θ*_(*x*) may be unreliable

This mismatch may lead to systematic errors, unpredictable reconstructions, or misinterpretation of subtle spectral features.^82–84^

Physics-informed ML introduces physical constraints directly into the learning process to mitigate such issues. Examples include enforcing intensity non-negativity, spectral smoothness, monotonicity over specific regions, or bounded curvature. These constraints act as regularizers, reducing the hypothesis space and preventing the model from learning unphysical mappings. Smoothness constraints, implemented *via* penalties on second-order derivatives, suppress artificial oscillations in reconstructed spectra. Non-negativity constraints prevent the model from generating physically impossible intensity values.

Physics-informed priors also enhance cross-instrument robustness. When models incorporate knowledge about optical response characteristics, noise structure, or expected spectral shapes, they become less sensitive to dataset bias.^85,86^ Hybrid architectures that combine mechanistic spectral equations with neural networks represent a promising direction. These models achieve interpretability while preserving the flexibility of data-driven learning.

Another important consideration is uncertainty quantification. Monte-Carlo dropout, ensemble methods, and Bayesian neural networks provide estimates of model confidence, helping identify predictions that may fall outside the learned data distribution. Altogether, incorporating physical constraints and uncertainty estimation strategies into ML frameworks ensures more reliable, stable, and generalizable spectral analysis—an essential requirement for real-world deployment.

## 4. Machine-learning-enhanced fluorescence signal processing of CQDs

### 4.1. ML-driven noise suppression and ultra-low-level signal recovery

ML has recently transformed the extraction of low-intensity fluorescence features from CQD-based sensors, particularly when analyte-induced spectral variations are nearly obscured by environmental noise. Conventional denoising and baseline-correction algorithms often fail when spectra contain subtle, nonlinear distortions produced by concentration-dependent quenching, ionic interference, or excitation instability.^19,76^ In contrast, machine-learning classifiers and regressors can characterize high-dimensional spectral manifolds and differentiate trace-level analytes from measurement noise. A representative advancement is the ML-enhanced CQD nanosensor framework where multiple supervised algorithms were trained on fluorescence signatures across analyte-free and analyte-present conditions, achieving discrimination of Cu^2+^ concentrations down to 100 pM, corresponding to a 10^6^-fold improvement in the functional limit of detection compared to the original material performance.^87^ This represents one of the strongest demonstrations that algorithmic reconstruction can exceed the intrinsic chemical sensitivity of the nanomaterial itself.

Despite these impressive enhancements, several methodological limitations persist. Many ML models rely on manually extracted features rather than end-to-end spectral learning, which reduce robustness when transferring to new laboratory conditions. Additionally, existing datasets remain relatively small, limiting generalizability under varying pH, ionic backgrounds, or illumination geometries. The lack of explicit modeling of excitation–emission coupling may also constrain performance when complex photophysical pathways are present.^21,42^ Further developments could include self-supervised learning to capture unlabeled spectral variability, physics-informed architectures embedding fluorescence kinetics, and domain adaptation techniques to mitigate batch-to-batch spectral drift. Overall, ML-based noise-domain discrimination has clearly demonstrated transformative improvements in LOD recovery, yet broader applicability will require methodological standardization, larger curated datasets, and rigorous cross-platform validation.^88^

### 4.2. ML-enabled signal enhancement and real-time intelligent fluorescence sensing in CQDs

Recent advances demonstrate that ML not only improves post-acquisition fluorescence data analysis but also fundamentally enhances the effective sensing performance of CQD-based nanosensors by enabling reliable signal recovery near the noise threshold and facilitating real-time analyte discrimination. In contrast to conventional chemometric approaches that rely on deterministic baseline correction and linear assumptions, ML-driven frameworks extract latent, high-dimensional spectral representations that remain inaccessible through traditional signal processing techniques, thereby effectively reducing the practical limit of detection (LOD) beyond the intrinsic capabilities of the nanomaterial system.^87^

For example, ML-enhanced CQD nanosensors have been successfully applied to heavy metal ion detection, where supervised learning models trained on fluorescence spectra enabled the discrimination of Cu^2+^ at concentrations as low as 100 pM. This corresponds to an improvement of up to 10^6^-fold relative to the original detection limit, while maintaining robust performance across multiple analytes, including Hg^2+^, Pb^2+^, Cr^6+^, and Fe^3+^. Importantly, these gains arise not from modifications in CQD chemistry, but from the algorithmic reconstruction of weak and noisy spectral signatures, highlighting the role of ML in redefining sensing sensitivity limits.^87^

Beyond single-analyte systems, ML-assisted CQD sensor arrays enable reliable multicomponent ion discrimination in complex aqueous environments. Stepwise prediction models combined with multivariate learning strategies allow simultaneous identification of multiple heavy metal ions, including Cr^6+^, Fe^3+^, Fe^2+^, and Hg^2+^, achieving classification accuracies of up to 95–100% even in highly interfered real water samples.^90^ This demonstrates the ability of ML to resolve highly collinear and overlapping spectral responses that cannot be effectively separated using conventional optical analysis.

In addition, ML-integrated CQD platforms have expanded into biosensing and biomedical diagnostics through portable and real-time architectures. For instance, dual-responsive CQDs combined with smartphone-based ML analysis enable simultaneous detection of cytosine and 5-methylcytosine in urine samples, providing accurate quantification of DNA methylation levels under real sample conditions.^91^ Similarly, lightweight architectures such as MobileViT have been employed for real-time Zn^2+^ detection in sweat using CQD fluorescence signals, achieving rapid inference and stable performance across diverse and interference-rich datasets.^88^

Overall, these studies indicate that ML integration in CQD fluorescence sensing operates at two complementary levels: (i) enhancement of signal extraction and reduction of effective detection limits, and (ii) enabling real-time, portable, and multi-analyte intelligent sensing in complex environments. This dual functionality highlights ML as a core analytical component rather than a simple post-processing tool in next-generation CQD-based sensing systems.

A clear distinction should be made between traditional fluorescence analysis and ML-enabled fluorescence signal processing in CQD-based sensing systems. Traditional approaches are primarily based on explicit assumptions of signal linearity, peak separability, and predefined noise correction procedures, typically employing methods such as baseline correction, peak fitting, and classical chemometric decomposition. These methods rely on explicit physical or statistical modeling of fluorescence signals and often struggle in CQD systems characterized by spectral overlap, excitation-dependent emission, and strong environmental variability. In contrast, ML-enabled fluorescence processing operates without predefined analytical assumptions and instead learns nonlinear mappings directly from data. This allows the extraction of latent spectral features, reconstruction of weak signals embedded in noise, and discrimination of highly overlapping emission patterns. Therefore, ML represents a paradigm shift from model-driven fluorescence analysis to data-driven signal inference in CQD sensing.

#### 4.2.1. Comparative analysis of ML paradigms in CQD fluorescence sensing

A systematic comparison of ML methodologies applied in CQD fluorescence sensing reveals that existing approaches differ significantly in terms of their functional roles, assumptions, and applicability across photophysically complex environments. Rather than a unified methodological framework, current literature reflects a task-dependent selection of algorithms driven primarily by dataset characteristics and spectral complexity.

Classical multivariate methods such as PCA, MCR-ALS, and related factorization techniques are primarily used for exploratory analysis and spectral decomposition. While PCA provides efficient dimensionality reduction, its linear assumption limits its capability to capture nonlinear excitation-dependent emission behavior in CQDs. In contrast, MCR-ALS and related constrained factorization approaches introduce partial physical interpretability by enforcing chemical constraints; however, they remain sensitive to initialization and spectral overlap, particularly in strongly coupled emission systems.

Ensemble learning models, including Random Forest and XGBoost, dominate classification and regression tasks in multi-analyte CQD sensing due to their robustness against noise, limited dataset size, and moderate spectral variability.^76,88^ These models are particularly effective in handling intensity-based and ratio-based features but lack explicit mechanisms to directly model wavelength-dependent spectral structures.

Deep learning architectures, especially convolutional neural networks (CNNs) and transformer-based models, provide superior capability for modeling nonlinear spectral relationships and long-range wavelength correlations. CNNs are effective in extracting localized spectral features such as peak shifts and curvature changes, while transformers are more suitable for global spectral pattern learning in high-dimensional fluorescence datasets. However, these advantages come at the cost of reduced interpretability and increased dependency on large, diverse training datasets.

From an application perspective, the suitability of ML methods in CQD fluorescence sensing is strongly scenario-dependent. Linear and chemometric methods are appropriate for low-complexity, well-controlled systems; ensemble methods are optimal for medium-complexity multi-analyte classification tasks; and deep learning approaches are most suitable for highly nonlinear, noisy, or real-time sensing environments. This indicates that no single ML paradigm is universally optimal; instead, method selection must be guided by photophysical complexity, dataset structure, and intended sensing application.^74–76^ Importantly, the differences outlined above directly govern the performance observed in CQD fluorescence sensing applications, particularly in terms of signal recovery at ultra-low analyte concentrations and discrimination of chemically similar species in multianalyte environments.

Across the reviewed CQD fluorescence sensing literature, a diverse set of ML algorithms has been employed, each serving a specific role in fluorescence signal processing and interpretation. In particular, Tian *et al.* applied seven different supervised learning models, including classical statistical classifiers and ensemble-based approaches, to extract weak fluorescence features near the noise threshold for heavy metal ion detection, demonstrating that ML can significantly enhance limit of detection (LOD) by reconstructing buried spectral signals rather than relying on direct intensity analysis.^87^ In a different context, PCA-based dimensionality reduction combined with multivariate curve resolution-alternating least squares (MCR-ALS) has been utilized to analyze large photoluminescence datasets and to interpret excitation-independent emission mechanisms in CQDs, providing both spectral decomposition and mechanistic insight into fluorescence behavior.^89^ For multianalyte classification, stepwise prediction models integrated with ML frameworks and ensemble strategies such as SX-models have been successfully implemented to distinguish multiple metal ions in complex water matrices with high accuracy, highlighting their robustness in noisy and overlapping spectral environments.^90^ Furthermore, deep learning approaches and lightweight neural architectures, including MobileViT-based models, have been introduced for real-time biosensing applications, enabling smartphone-based fluorescence analysis and improving classification accuracy in biological samples.^88,91^ Collectively, these studies demonstrate that ML algorithms in CQD fluorescence sensing range from classical multivariate statistical methods (PCA, MCR-ALS), to ensemble learning models, and advanced neural networks, with each category addressing different levels of spectral complexity, noise conditions, and application requirements.

#### 4.2.2. Mechanistic insights and technical barriers in ML-enhanced CQD sensing

From a mechanistic standpoint, the role of ML extends beyond statistical pattern recognition and directly addresses fundamental photophysical constraints inherent to CQD systems. CQDs typically exhibit heterogeneous emissive states, including surface traps, defect-related recombination centers, and excitation-dependent radiative pathways, which result in broadened and overlapping fluorescence spectra. In this context, ML models function as inverse mapping operators that reconstruct latent signal structures from distorted and noisy observations. This enables partial compensation for intrinsic limitations such as non-uniform emission profiles, low quantum yield variability, and environmental perturbations, without altering the underlying photophysical processes. Therefore, ML should be viewed as a computational layer that enhances signal interpretability and measurement reliability rather than modifying the material's intrinsic optical behavior.

While recent studies demonstrate significant improvements in ultra-low concentration detection and multi-analyte classification using ML-enhanced CQD fluorescence systems, a deeper mechanistic interpretation reveals that these performance gains originate from specific signal processing transformations rather than purely material-based enhancements.^87,88^

In ultra-low concentration analyte detection, the primary ML-driven advantage lies in the ability of algorithms to reconstruct weak fluorescence signatures embedded within stochastic noise. Classical detection approaches assume relatively stable signal-to-noise separation; however, CQD fluorescence signals are often affected by photon fluctuation, environmental interference, and excitation instability. ML models, particularly supervised classifiers and ensemble learning methods, learn high-dimensional decision boundaries that effectively map subtle nonlinear variations in spectral distributions. This allows discrimination of analyte-induced perturbations even when the signal intensity is below conventional detection thresholds. However, this improvement is fundamentally constrained by the quality and diversity of training datasets, and models may fail under domain shifts such as changes in solvent composition, excitation source, or CQD surface chemistry.

In multi-analyte classification systems, the improved performance arises from the ability of ML algorithms to exploit correlated spectral features across multiple wavelength channels. Instead of relying on single peak shifts, ML models integrate global spectral patterns, intensity ratios, and curvature-based descriptors to construct separable feature spaces for chemically similar ions. This is particularly important in CQD-based sensors, where overlapping emission pathways and broad spectral bands prevent conventional deconvolution methods from achieving reliable discrimination. Nevertheless, this advantage is accompanied by a key limitation: as analyte complexity increases, spectral collinearity becomes stronger, leading to feature entanglement and reduced classification confidence without proper regularization or uncertainty quantification.

From a mechanistic perspective, both applications are fundamentally governed by the interaction between CQD photophysical variability and data-driven pattern recognition. ML does not physically enhance emission efficiency; rather, it improves the effective extractability of information from noisy and overlapping spectral signals. This distinction is critical for correct interpretation of reported performance enhancements such as ultra-low detection limits or near-perfect classification accuracy.^81,89^

Despite these advances, several technical barriers remain unresolved. First, lack of physically constrained learning can lead to overfitting and poor generalization across different experimental setups. Second, limited availability of large, standardized spectral datasets restricts model robustness. Third, most current approaches lack explicit uncertainty quantification, which is essential for real-world analytical deployment. Finally, the absence of integrated photophysical-computational models limits the interpretability of ML outputs in terms of CQD emission mechanisms such as defect-state transitions, surface trap dynamics, and excitation-dependent emission behavior.

Overall, while ML significantly enhances the practical performance of CQD-based fluorescence sensing systems, its role should be interpreted as an information extraction and pattern recognition tool rather than a direct modifier of underlying photophysical processes. A more rigorous integration of physical modeling and data-driven learning is therefore required to overcome current methodological limitations and enable reliable real-world applications.

A critical aspect in CQD fluorescence sensing is the robustness of ML models under low signal-to-noise ratio (SNR) conditions, where fluorescence signals are significantly distorted by photon noise, environmental fluctuations, and instrumental instability. In such scenarios, traditional signal processing methods fail to reliably extract meaningful spectral features due to the dominance of stochastic noise components. ML-based approaches, however, improve robustness by learning high-dimensional feature representations that can partially separate weak analyte-induced signals from background noise. This enables improved detection performance even near the noise threshold, as demonstrated in heavy metal ion sensing where ML models successfully identify signals otherwise indistinguishable from noise.^88,90^ However, robustness is not absolute, as model performance strongly depends on training dataset quality, spectral diversity, and representativeness of noise conditions. In cases of domain shift—such as changes in excitation wavelength, solvent environment, or CQD surface chemistry—model generalization may degrade, leading to reduced accuracy and potential misclassification. Therefore, while ML significantly enhances signal extraction under low SNR conditions, its robustness is fundamentally constrained by dataset bias and experimental variability. Addressing these limitations requires inclusion of diverse training spectra, noise augmentation strategies, and integration of physically informed constraints to improve generalization across real-world sensing environments.

### 4.3. Spectral decomposition and photoluminescence mechanism interpretation *via* ML

ML-assisted decomposition of CQD, PL spectra has emerged as a powerful analytical framework for resolving broad, asymmetric, and highly overlapping emission bands arising from surface functional groups, defect states, and excitation-dependent recombination pathways. Traditional spectral deconvolution approaches, such as Gaussian fitting and classical chemometric models, rely on predefined functional assumptions and linear separability, which are often insufficient to capture the intrinsic complexity of CQD emission behavior under varying excitation and environmental conditions.^87,90^

To overcome these limitations, multivariate and data-driven ML techniques such as principal component analysis (PCA), multivariate curve resolution-alternating least squares (MCR-ALS), and non-negative matrix factorization (NMF-ARD-SO) have been widely adopted for spectral decomposition tasks. These methods enable the extraction of latent spectral components from high-dimensional fluorescence datasets, allowing identification of hidden emissive states and subtle spectral variations that are otherwise obscured in raw PL signals. For instance, PCA-based decomposition has been used to reduce spectral dimensionality and reveal clustering behavior associated with excitation-dependent emission profiles, while MCR-ALS provides chemically interpretable components under constrained optimization frameworks.^89^

More advanced factorization approaches, particularly NMF-ARD-SO, introduce automatic model order selection, enabling the identification of the optimal number of physically meaningful emission components without subjective bias. In CQD systems, such approaches have revealed that certain nanostructures exhibit a dominant single-state emission mechanism rather than a multi-state superposition, challenging previously accepted interpretations of CQD photophysics.^89^ This represents a significant advancement in linking computational decomposition with physical emission mechanisms.

In addition to mechanistic interpretation, ML-based spectral decomposition has demonstrated practical relevance in sensing applications. In heavy metal ion detection systems, learned spectral features extracted from noisy fluorescence backgrounds enable robust discrimination of analytes at ultra-low concentrations by enhancing signal separability near the detection limit.^87^ Similarly, in multianalyte environmental sensing, ML-assisted frameworks improve classification accuracy by resolving overlapping spectral signatures in complex aqueous matrices, even under strong interference conditions.^90^ Furthermore, lightweight neural and smartphone-integrated ML systems extend these capabilities to real-time biosensing and portable analytical platforms, enabling direct translation of spectral patterns into quantitative outputs.^88,91^

Despite these advances, several limitations remain. Classical methods such as PCA and MCR-ALS are sensitive to noise, require manual selection of components, and may not always yield physically unique solutions. Moreover, most existing studies are limited to steady-state PL spectra, while time-resolved and dynamic emission processes remain underexplored in ML-based frameworks. Environmental drift, excitation variability, and synthesis-dependent heterogeneity further reduce the robustness of decomposition models across different CQD systems.

A critical aspect of ML-based CQD fluorescence analysis is the physical interpretation of extracted features in relation to underlying photophysical properties. In CQD systems, ML-derived features such as principal components, latent vectors, and spectral loadings are not purely mathematical constructs but often encode physically meaningful information associated with surface defect states, trap-mediated recombination, and carrier relaxation dynamics. For instance, variations captured in PCA score space can reflect excitation-dependent emission shifts arising from heterogeneous surface energy levels, while MCR-ALS and NMF-derived components can correspond to distinct emissive contributions linked to core and surface recombination pathways. Similarly, intensity ratios and curvature-based features used in supervised learning models are often influenced by charge transfer processes and environmental modulation of surface functional groups. In heavy metal ion sensing systems, ML feature spaces effectively capture perturbations in carrier dynamics induced by analyte-CQD interactions, thereby indirectly encoding changes in non-radiative recombination rates and surface binding effects. However, it is important to note that these correlations are typically implicit rather than explicitly enforced, and therefore require careful post hoc interpretation supported by photophysical understanding. This highlights the necessity of combining ML-based feature extraction with mechanistic CQD models to ensure physically meaningful interpretation of learned representations.

Panels 4A and 4B demonstrate how PCA organizes the multidimensional photoluminescence dataset of the CQDs into a reduced feature space where excitation- and pH-dependent variations become clearly distinguishable. Upon projection onto the first two principal components, three well-defined clusters emerge, each corresponding to characteristic emission responses of the CQDs under different excitation regimes and mild environmental changes. This separation highlights the ability of PCA to extract the dominant variance sources—such as excitation-specific recombination behavior and subtle pH-induced spectral shifts—that are masked within the broad, overlapping PL profiles typical of CQDs. The resulting score distribution confirms that PCA, despite its linear nature, can effectively uncover latent structure and chemically relevant trends, providing an unbiased view of spectral heterogeneity in CQD emission matrices. Thus, Panels A and B underline PCA's dual role as both a compression tool and a mechanistically informative mapping method for CQD photophysics.

**Fig. 4:**
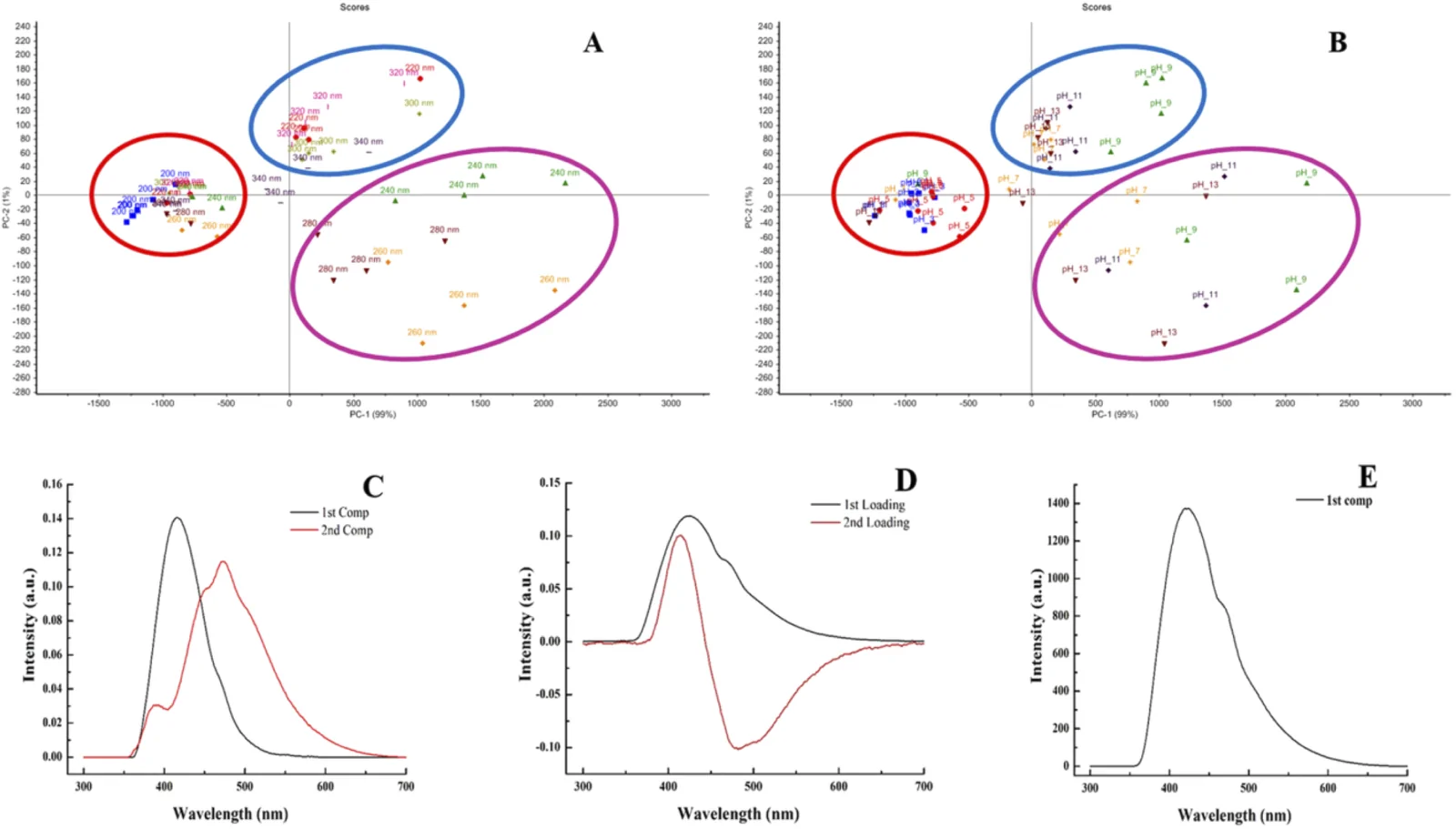
Multivariate analysis of the photoluminescence behavior of the CQDs. (A and B) PCA score maps revealing excitation- and pH-dependent clustering. (C and D) Spectral decomposition using MCR-ALS and PCA loadings, showing overlapping and partially non-physical components. (E) NMF-ARD-SO identifying a single non-negative component, indicating a unified emissive state in the CQDs. Adapted with permission from ref. 89. © 2019 Nature.

Panels 4C and 4D investigate how linear spectral-decomposition methods behave when applied to the broad PL band of the CQDs, revealing both their interpretational potential and their inherent limitations. MCR-ALS resolves the CQD emission into two partially overlapping components at 416.2 and 475.3 nm, whereas PCA loadings yield two vectors, one displaying a negative amplitude that undermines its physical interpretability. Such overlap and sign ambiguity indicate that linear methods may generate mathematically valid but physically unreliable components when CQD emission arises from strongly coupled or highly overlapped transitions. In contrast, Panel 4E highlights the capability of NMF-ARD-SO to identify the true spectral dimensionality: the algorithm converges to a single non-negative component centered at 424.7 nm, faithfully reconstructing the original CQD PL band. By eliminating redundant basis functions through automatic relevance determination, the method reveals that the asymmetric emission originates from an intrinsic single-state transition rather than multiple emissive pathways. Collectively, Panels C–E show that constraint-aware factorization provides a more physically grounded interpretation of CQD photophysics (Fig. 4).

### 4.4. Multichannel fluorescence feature extraction and multi-analyte classification

Fluorescence sensor arrays based on CQDs increasingly rely on ML to extract discriminative features from complex multichannel spectra, especially in cases where analytes induce overlapping or nonlinear spectral shifts. In recent work, stepwise-prediction-assisted ML methods enabled simultaneous quantification and classification of multiple metal ions, including Cr^6+^, Fe^3+^, Fe^2+^, and Hg^2+^, across mixed solutions and real environmental samples, achieving accuracies up to 100% for field-collected lake water. The use of multichannel feature fusion allowed the ML model to recognize subtle combinations of intensity ratios and spectral curvature patterns that traditional chemometric methods misclassify. More importantly, the model demonstrated resilience to matrix interferences, indicating that robust ML workflows can outperform material-limited selectivity by compensating algorithmically for spectral overlap.^90^

Despite these strengths, current approaches still face methodological challenges. Most models employ classical algorithms requiring predefined features, which may overlook nonlinear spectral interactions inherent in CQD systems. Additionally, the reliance on laboratory-calibrated training data can hinder model transferability across different optical instruments, sample containers, or environmental backgrounds.^26,42^ The generalization to extreme concentration ranges or highly contaminated matrices is also insufficiently explored. Another important limitation is the lack of uncertainty quantification; ML classifiers typically provide deterministic outputs with no confidence estimation, complicating risk-sensitive applications such as environmental monitoring. Future research should incorporate deep spectral encoders, probabilistic classifiers, and domain generalization strategies. Integration of physics-based fluorescence constraints may further reduce overfitting and improve reliability.^91^ Nevertheless, the evidence to date demonstrates that ML-driven multichannel feature extraction fundamentally enhances both single- and multi-analyte sensing performance, especially when analyte-induced spectral signatures are subtle or partially collinear.

**Fig. 5:**
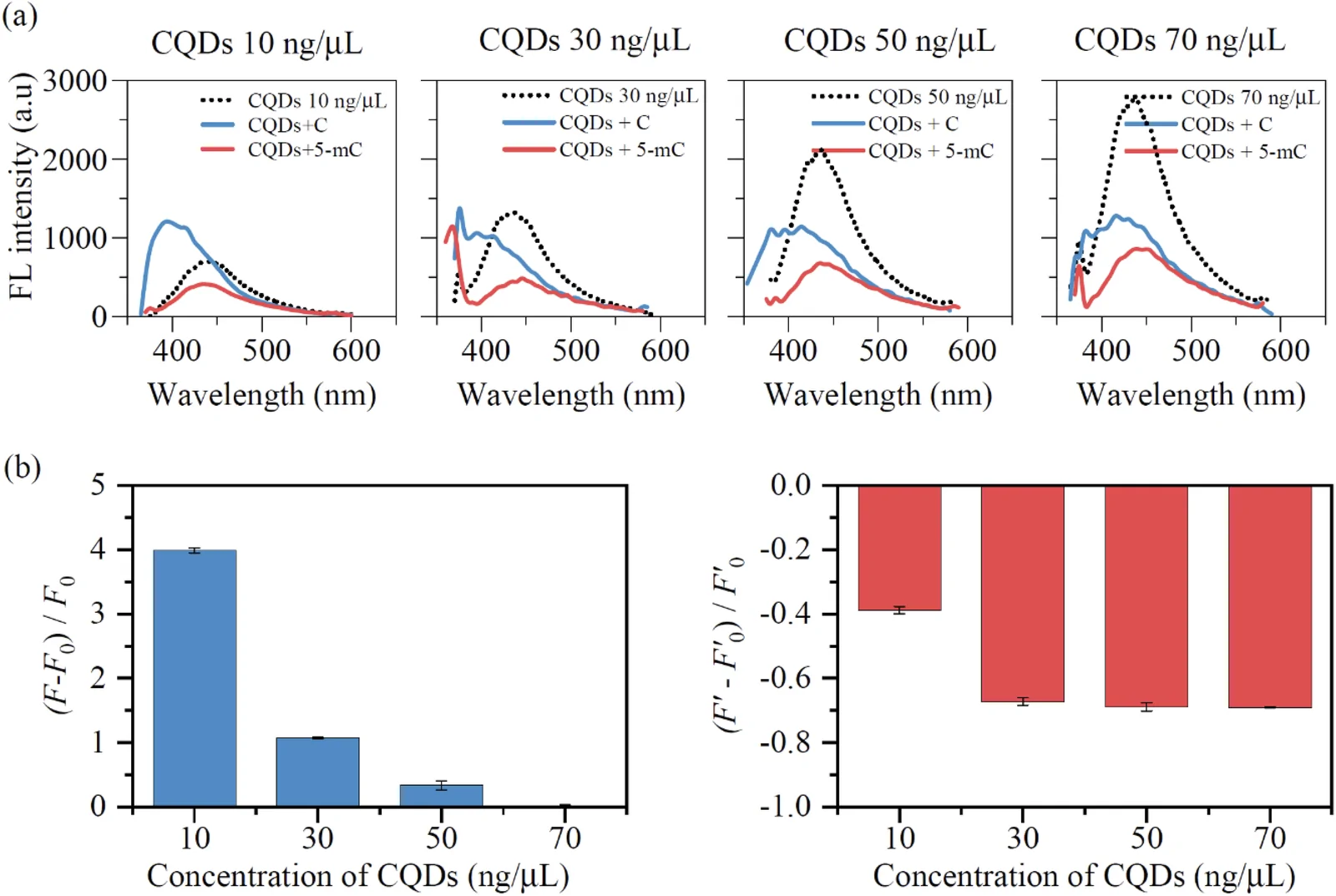
Multichannel fluorescence responses of CQDs at varying concentrations upon the addition of cytosine © and 5-methylcytosine (5-mC). (a) Emission spectra highlighting concentration-dependent enhancement and quenching patterns. (b) Relative fluorescence changes, providing discriminative multichannel signatures for analyte differentiation. Adapted with permission from ref. 91. © 2023 American Chemical Society.

Fig. 5 illustrates how multichannel fluorescence responses of the CQDs encode analyte-specific information that can be leveraged for machine-learning-based feature extraction. In panel (a), the emission spectra of CQDs at four concentrations (10–70 ng µL^−1^) show distinct and concentration-dependent modulation patterns upon the addition of cytosine © or 5-methylcytosine (5-mC): C induces a pronounced fluorescence enhancement, whereas 5-mC generates systematic quenching across the entire spectral band, with each concentration producing a unique combination of peak reshaping and relative-intensity redistribution. These nonlinear and partially overlapping spectral signatures exemplify exactly the type of complex multichannel variation that classical chemometric tools fail to separate reliably. Panel (b) quantifies these effects through relative enhancement (left) and relative quenching (right), revealing concentration-dependent contrast that remains consistent but nonlinearly scaled, highlighting the presence of multidimensional spectral cues beyond simple intensity changes. Such structured variations—arising simultaneously from concentration, base identity (C *vs.* 5-mC), and spectral curvature—provide an ideal foundation for ML-driven feature extraction, where fused intensity ratios and multichannel patterns allow robust discrimination even under spectral overlap. The figure therefore demonstrates how CQD sensor arrays inherently generate rich fluorescence fingerprints that can be algorithmically mined to classify chemically similar analytes and potentially support multi-analyte sensing in more complex environments.

### 4.5. Real-time intelligent fluorescence sensing and next-generation CQD signal processing

Real-time processing of CQD fluorescence signals has progressed significantly due to recent advances in lightweight ML architectures, enabling mobile, on-device inference for practical biochemical sensing. A prominent example integrates spectral feature clustering with a compact MobileViT-based classifier to detect Zn^2+^ in sweat, reaching an LOD of 0.17 µM and an accuracy of 82.4% across more than 650 fluorescence measurements.^88^ This architecture demonstrates how deep learning can be optimized for embedded applications, enabling fast quantification without reliance on benchtop spectrofluorometers. Such systems highlight an important trend: the shift from laboratory-dependent fluorescence readouts toward decentralized and real-time sensing using computational enhancement to overcome hardware limitations. A similar transition appears in mobile-based detection of cytosine and 5-methylcytosine, where machine-learning-assisted interpretation of excitation-dependent CQD fluorescence enabled accurate quantification of DNA methylation levels directly from urine samples.^91^ These developments illustrate how ML can transform CQD fluorescence from a passive analytical readout into an active, self-correcting sensing platform.

Yet several critical issues remain underexplored. The stability of ML performance under variable illumination conditions, sensor aging, and user-dependent variations is insufficiently evaluated. Additionally, mobile models often rely on down-sampled or compressed spectra, potentially omitting diagnostic high-frequency features. The interpretability of deep feature maps also remains a barrier to regulatory acceptance in biomedical applications.^21,42^ Finally, the integration of ML workflows with photophysical modeling is still minimal; models rarely incorporate knowledge about electron-transfer pathways, defect-state lifetimes, or Förster-type interactions. Future directions include adaptive calibration, federated learning across distributed devices, and multimodal fusion with electrochemical or thermal data. Overall, real-time ML-enabled fluorescence processing marks a significant evolution in CQD-based sensing and represents a key driver toward next-generation high-accuracy detection systems.^88,91^

### 4.6. Hybrid physics-informed and data-driven modeling for mechanistically interpretable CQD fluorescence analytics

Recent advances in ML have created new opportunities for integrating physics-informed constraints with data-driven fluorescence modeling to enhance both interpretability and accuracy in CQD-based sensing systems. While traditional ML approaches such as supervised classifiers for ultra-low-level metal detection,^87^ matrix factorization for PL mechanism elucidation,^89^ and multichannel prediction for complex ion mixtures^90^ have demonstrated impressive performance, they often operate as black boxes that do not explicitly embed the underlying photophysical processes governing CQD emission. Physics-informed ML (PI-ML) seeks to overcome this limitation by incorporating mechanistic priors reflecting excitation-dependent transitions, defect-state recombination, and photoinduced electron-transfer pathways into the learning architecture. This creates hybrid models that constrain the solution space to physically plausible behaviors, improving robustness against noise, instrument difference, and environmental drift.

A mechanistically grounded framework is particularly valuable for CQD fluorescence due to its inherent nonlinearities. Excitation-dependent shifts, multi-state surface recombination, and pH-driven modulation of emissive sites—as exemplified in integrated PL datasets analyzed *via* PCA, MCR-ALS, and NMF-ARD-SO^89^—often produce overlapping or asymmetric peaks that challenge purely data-driven decomposition. Embedding photophysical constraints (*e.g.*, non-negativity, wavelength-dependent smoothness, or energy-state continuity) could drastically improve model stability and provide more reliable spectral reconstructions. Furthermore, PI-ML could enhance discriminative tasks where analyte-induced fluorescence changes partially overlap with environmental noise, a challenge observed in Cu^2+^ detection at concentrations as low as 100 pM^87^ and in complex multi-ion classification frameworks.^90^

These hybrid models also align closely with the emerging trend of mobile and resource-constrained device-based sensing. For example, smartphone-integrated classification of cytosine and 5-methylcytosine relies on compact architectures trained on fluorescence images of nitrogen-doped CQDs,^91^ and MobileViT-based inference enables real-time Zn^2+^ detection in sweat.^88^ Embedding physics-informed layers in these lightweight networks could reduce data requirements and improve generalization under variable illumination or sample conditions. Future development pathways include differentiable photophysical simulators, PI-regularized spectral encoders, and hybrid inverse-forward reconstruction models. Overall, PI-ML offers a compelling direction for mechanistically interpretable, accurate, and resource-efficient CQD fluorescence sensing.

ML interpretability in CQD fluorescence sensing plays a critical role in linking data-driven predictions to underlying photophysical mechanisms. While advanced models such as deep neural networks and ensemble classifiers provide high predictive accuracy, their outputs must be interpreted in terms of CQD emission physics to ensure scientific validity. In this context, feature-based interpretability methods such as principal component analysis (PCA) and multivariate curve resolution (MCR-ALS) enable partial physical mapping of fluorescence signals by associating latent variables with excitation-dependent emission behavior, surface defect states, and trap-mediated recombination pathways. Similarly, feature importance analysis and latent space visualization in supervised learning models allow identification of wavelength regions that contribute most significantly to analyte discrimination. Importantly, these interpretable representations provide a bridge between abstract mathematical learning spaces and physically meaningful CQD photophysics. However, full interpretability remains limited in deep learning models due to nonlinear feature entanglement, highlighting the need for physics-informed constraints to ensure that learned representations remain consistent with emission mechanisms such as surface recombination dynamics and defect-state transitions.

Physics-informed machine learning (PI-ML) provides a crucial bridge between purely data-driven fluorescence analysis and the fundamental photophysical mechanisms governing CQD emission. Unlike conventional ML models that rely solely on statistical correlations, PI-ML frameworks incorporate physical constraints derived from CQD photophysics, including excitation-dependent emission, surface defect-state recombination, quantum confinement effects, and energy transfer dynamics. By embedding these constraints into the learning process, the model is guided toward physically consistent solution spaces, reducing the risk of spurious correlations and improving generalization across different experimental conditions. In CQD fluorescence systems, this approach is particularly important because spectral responses are governed by strongly nonlinear and environment-sensitive processes that cannot be fully captured by purely empirical models.^87,89^ Physics-informed architectures, such as constrained neural networks and hybrid mechanistic-data-driven models, allow fluorescence reconstruction to remain consistent with known emission pathways while still benefiting from the flexibility of machine learning. Consequently, PI-ML serves as a conceptual and computational bridge, enabling not only improved predictive performance but also enhanced physical interpretability of learned representations in CQD sensing systems.

### 4.7. Generative and augmentation-driven data expansion for high-accuracy CQD fluorescence sensing

A major limitation of current CQD-based fluorescence sensing is the scarcity of high-quality, high-diversity spectral datasets. Most existing studies rely on limited, single-laboratory measurements: *e.g.*, Cu^2+^ and multi-metal detection using ML-enhanced spectra, mechanistic PL decomposition of excitation-pH matrices, multi-ion discrimination across lake water samples, molecular biomarker sensing *via* smartphone-assisted CQDs, and real-time Zn^2+^ quantification in sweat using mobile deep learning.^87,90^ These datasets, while valuable, do not capture the full variability of CQD synthesis methods, environmental perturbations, nor device-dependent noise signatures. As a result, ML models often exhibit impressive in-domain accuracy yet limited generalizability.

Generative modeling—particularly variational autoencoders (VAEs), diffusion models, and adversarial spectral generators—offers a promising direction to address this constraint by synthesizing realistic fluorescence spectra that emulate excitation-dependent behavior, peak asymmetry, quenching dynamics, and multichannel variability. Such models could generate augmented training sets reflecting conditions that are difficult, expensive, or chemically unstable to reproduce experimentally. For instance, synthetic spectra could simulate extremely low Cu^2+^ concentrations near the noise boundary, complementing the high-performance models that achieved 10^6^-fold LOD enhancement. Similarly, generative augmentation could reproduce subtle PL shifts across pH and excitation ranges observed in integrated CQD datasets, or simulate mixtures of metal ions with overlapping features to support multi-analyte classifiers.^88,91^

In smartphone-assisted CQD sensing, generative augmentation could compensate for variable lighting, camera calibration differences, and heterogeneous urine backgrounds in cytosine/5-methylcytosine detection. Likewise, mobile Zn^2+^ sensing could benefit from ensembles of augmented spectra representing sweat composition variability and competing interferents.^88^

A key advantage of generative data expansion is the ability to produce balanced datasets that reduce class imbalance—common in trace-level and multi-analyte applications—while enabling more robust uncertainty modeling. Nonetheless, several challenges remain, including the risk of generating physically implausible spectra, mode collapse, and overfitting to synthetic data distributions. Future developments should prioritize physics-guided generative constraints, domain adversarial validation pipelines, and hybrid real-synthetic training strategies. Generative augmentation thus stands out as a transformative tool for building scalable, generalizable, and highly accurate CQD fluorescence sensing frameworks.

### 4.8. Industrial translation and scale-up challenges of ML-enhanced CQD fluorescence sensing

Despite substantial advances in ML-enhanced CQD fluorescence sensing at the laboratory scale, the transition toward industrial deployment and large-scale manufacturing remains limited by a set of system-level engineering and scalability constraints that extend beyond algorithmic performance or sensing accuracy.

One of the most critical challenges is the lack of standardized large-scale CQD production protocols. In laboratory settings, CQDs are typically synthesized under tightly controlled conditions, resulting in optimized optical properties for specific experimental batches. However, scaling these processes to industrial levels introduces significant variability in precursor quality, reaction kinetics, temperature gradients, and surface passivation efficiency. These factors lead to batch-to-batch inconsistency in emission profiles, quantum yield distribution, and surface defect density. Such variability directly undermines the reliability of ML models that assume stable and reproducible input feature distributions.

Another key limitation arises from the absence of standardized calibration and validation protocols across different sensing platforms. In industrial environments, CQD-based sensors must operate across heterogeneous optical systems, detectors, and excitation sources. Variations in optical path length, detector sensitivity, and excitation wavelength stability introduce systematic deviations in fluorescence readouts, making direct transfer of trained ML models between instruments highly unreliable without extensive recalibration or domain adaptation.^88,90^

Furthermore, real-world deployment introduces challenges associated with operational robustness and long-term stability. CQD-based sensing systems are often exposed to fluctuating environmental conditions such as temperature variation, humidity, and photobleaching effects, all of which can gradually alter fluorescence response characteristics. In the absence of adaptive recalibration mechanisms, ML model performance may degrade over time, limiting their suitability for continuous monitoring applications.

From an integration perspective, another barrier is the lack of unified hardware–software co-design strategies. Most existing systems treat CQDs as passive sensing materials and ML algorithms as independent post-processing tools. This separation restricts system optimization, particularly in embedded or portable devices where computational resources, power consumption, and real-time processing constraints must be jointly considered. The absence of optimized edge-computing frameworks further limits deployment in field-based or point-of-care scenarios.^48,91^

Finally, regulatory and standardization gaps also hinder commercialization. There is currently no universally accepted benchmark for evaluating CQD-based ML sensing systems in terms of accuracy, stability, reproducibility, and cross-platform compatibility. Without such standards, industrial adoption remains fragmented and application-specific rather than scalable. Overall, overcoming these challenges requires not only advances in material synthesis and ML algorithm design, but also the development of standardized production protocols, cross-platform calibration frameworks, and integrated sensing architectures that jointly address material variability, system stability, and deployment constraints.

### 4.9. Critical assessment of assumptions, limitations, and reliability constraints in ML-enabled CQD fluorescence sensing

Despite the remarkable advances of ML in enhancing CQD fluorescence sensing performance, a critical evaluation of the underlying assumptions and methodological constraints is essential for realistic assessment of its applicability. Most ML frameworks employed in CQD-based sensing implicitly assume that training and testing data originate from similar statistical distributions. However, this assumption is frequently violated in practical fluorescence environments due to variations in CQD synthesis routes, surface functionalization, excitation conditions, solvent effects, and instrumental configurations. Such variability leads to domain shift, which can significantly degrade model generalization and reduce predictive reliability when applied to unseen experimental conditions.

Another fundamental limitation arises from data dependency. Supervised ML models require large, diverse, and accurately labeled fluorescence datasets to achieve robust performance. In CQD systems, however, data acquisition is often constrained by experimental noise, limited reproducibility, and time-dependent photophysical instability, including photobleaching and surface state fluctuations. These factors restrict dataset scalability and introduce bias into model training, potentially resulting in overfitting to narrow experimental conditions.^72,88^

Model interpretability also represents a significant challenge. Advanced architectures such as deep neural networks and transformer-based models function as complex nonlinear mapping systems, often operating as “black-box” predictors. While these models provide high accuracy in classification and regression tasks, they do not inherently offer physically interpretable relationships between fluorescence signals and underlying photophysical mechanisms, such as defect-state emission, exciton recombination, or surface trap dynamics. This limits their utility in mechanistic studies of CQDs.

Furthermore, ML models may inadvertently learn spurious correlations in spectral data, particularly under low signal-to-noise ratio (SNR) conditions, where noise patterns can be misinterpreted as meaningful features. This issue becomes more pronounced in multianalyte sensing scenarios, where spectral overlap increases the risk of feature entanglement and misclassification. In addition, most current ML approaches assume temporal stability of fluorescence signals, whereas CQD systems often exhibit time-dependent variations due to environmental drift and excitation instability.

Addressing these limitations requires the integration of physics-informed constraints, improved dataset standardization, uncertainty quantification, and cross-domain validation strategies.^69,89^ Hybrid frameworks combining photophysical modeling with data-driven learning are expected to improve robustness and ensure reliable deployment in real-world CQD fluorescence sensing applications.

## 5. Future frontiers: toward autonomous, intelligent, and mechanistically-aware CQD fluorescence sensing

### 5.1. Autonomous closed-loop CQD fluorescence platforms integrating real-time spectroscopy, ML feedback, and adaptive control systems

The next generation of CQD fluorescence sensing systems will be defined by a transition from passive readout architectures toward fully autonomous closed-loop platforms. Instead of collecting spectra and subsequently processing them offline, future systems will integrate synchronized modules for automated excitation control, real-time signal interpretation, and dynamic adjustment of acquisition parameters based on algorithmic assessment of spectral quality. Such architectures represent a paradigm shift: they no longer treat ML as a post-processing tool but rather as the central orchestrator governing the entire photophysical interrogation pipeline.

In these intelligent systems, ML models will quantify spectral uncertainty, identify emerging drift, and autonomously trigger an adjustment in excitation wavelength, integration time, gain profile, or background normalization to optimize fluorescence detectability on-the-fly. For instance, when detection occurs near the noise floor—common for trace Cu^2+^, multimetal mixtures, or low-abundance biochemical markers—the system could automatically increase spectral resolution, adopt alternative excitation wavelengths, or invoke a denoising module to maintain analytical reliability. Such adaptive loops would be particularly powerful when dealing with ambient variations such as pH fluctuations, solvent composition changes, variable sweat composition, or dynamic interactions in complex biofluids.

An equally transformative frontier involves the integration of closed-loop ML with active photonic elements. Tunable LEDs, acousto-optic filters, and micro-spectrometers could continuously adjust optical pathways based on incoming signal morphology. Future CQD sensors may incorporate miniaturized photonic circuits that dynamically lock onto optimal emission bands through feedback-based wavelength tracking. This would allow devices to follow analyte-induced PL shifts even under rapidly changing chemical environments.

The shift toward fully autonomous sensing also unlocks opportunities for self-calibrating systems. Such platforms could compensate for photobleaching, surface-state instability, or instrumental drift by continually retraining lightweight local models or uploading model gradients to cloud-based federated networks. Decentralized calibration will be crucial for wearable and point-of-care devices, which often encounter inconsistent environmental conditions and variable user-dependent handling.

Over the next decade, these closed-loop, real-time spectroscopic systems will evolve toward complete autonomy: performing spectral acquisition, drift correction, feature extraction, anomaly detection, and decision making without human intervention. As the field converges on autonomous control, CQD sensing will expand from laboratory setups into distributed, intelligent, and adaptive platforms capable of accurate operation in uncontrolled real-world environments.

### 5.2. Mechanistically-grounded and multimodal ML architectures unifying CQD photophysics, chemical interactions, and cross-domain spectral behavior

A second major frontier involves the development of ML architectures that are deeply intertwined with the mechanistic foundations of CQD photophysics. Traditional models—including supervised classifiers, matrix factorization approaches, and mobile inference engines—have been effective for tasks such as denoising, ultra-low-level detection, spectral decomposition, and multianalyte classification. However, current ML pipelines rarely account for the complexities of CQD emission mechanisms, including the coexistence of surface and core states, defect-mediated recombination, photoinduced electron transfer, dopant-induced energy redistribution, and excitation-pH coupling.

Future architectures will move beyond empirical correlations by explicitly embedding photophysical constraints into their learning objectives. Emerging paradigms such as physics-informed neural networks (PINNs), differentiable fluorescence simulators, and spectral-kinetic hybrid models can integrate quantum yield dependencies, Förster resonance coupling, wavelength-dependent scattering, and exciton–phonon interactions directly into the network structure. By constraining the hypothesis space, these models will generalize more efficiently across diverse CQD synthesis batches, environmental backgrounds, and instrument architectures.

Another critical direction involves the fusion of multimodal data streams. Next-generation CQD sensors will increasingly operate at the intersection of fluorescence spectroscopy, electrochemical transduction, Raman scattering, temperature sensing, or impedance monitoring. Multimodal fusion networks combining these signals will enable richer characterization of analyte behavior, distinguish spectral overlaps that are difficult to resolve in purely optical domains, and improve mechanistic interpretability.

Furthermore, the integration of real-time temporal components will be essential. Current studies emphasize steady-state spectra, but next-generation ML models will incorporate time-resolved PL trajectories, blinking patterns, transient quenching dynamics, and lifetime-based contrast mechanisms. Temporal attention networks and recurrent spectral encoders could capture these photophysical evolutions, enabling finer discrimination near LOD boundaries and offering deeper insight into analyte-CQD energy transfer pathways.

In addition, domain generalization and transfer learning architectures will become fundamental for ensuring robust performance across platforms. Models trained on datasets collected at specific excitation wavelengths, sample matrices, or synthesis conditions must be capable of adapting to new environments without full retraining. Domain-invariant spectral encoders, adversarial alignment methods, and few-shot adaptation strategies will allow fluorescence models to maintain high accuracy across laboratories, measurement systems, and environmental conditions.

The long-term vision is a unified ML-photophysics framework capable of capturing the chemical, structural, spectral, and temporal behavior of CQDs in a single interpretable architecture. Such models will bridge the gap between fundamental quantum photophysics and applied sensing, enabling a new generation of highly reliable, mechanism-aware fluorescence systems.

### 5.3. Scalable, distributed, and user-integrated ecosystems for global CQD sensing: from personalized health to environmental intelligence

The future landscape of CQD fluorescence sensing extends far beyond laboratory applications and will be shaped by the development of distributed, interconnected sensing ecosystems. These ecosystems will support applications ranging from personalized biochemical monitoring and precision nutrition to environmental intelligence, water quality surveillance, and industrial process analytics. Unlike traditional bench-scale setups, the next wave of systems will rely on networks of low-cost, portable, and user-operated fluorescence devices augmented by cloud-based intelligence and machine-learning-driven harmonization.

At the personal health level, CQD-based biosensors integrated with smartphones and wearables will enable continuous monitoring of biomarkers such as trace metals, stress-related metabolites, oxidative stress indicators, and epigenetic markers. ML-driven interpretation layers will compensate for variations in sample volume, sweat rate, urine composition, or photophysical drift, allowing robust quantification even under unstructured human-centric conditions. Over time, devices may incorporate personalized calibration models that evolve based on each user's biochemistry and produce individualized baselines for health management.

At the community and environmental levels, distributed fluorescence nodes will form collaborative networks for large-scale monitoring of water sources, agricultural runoff, industrial discharges, and heavy-metal contamination. These networks could leverage federated learning to combine insights from heterogeneous sensors without sharing raw data, preserving privacy and security while building globally calibrated spectral models. Such harmonization ensures that measurements collected using different CQD syntheses, container materials, or portable spectrometers remain interoperable.

The next generation of platforms will also integrate automated anomaly detection to identify emergent patterns indicative of contamination events or ecosystem disturbances. ML-enabled spectral drift diagnostics could detect early signs of sensor degradation or biofouling, prompting recalibration or replacement before performance declines. Over time, this will transition environmental monitoring from reactive sampling to predictive, preventive intelligence.

Industrial and pharmaceutical applications represent another major frontier. CQD-based fluorescence monitoring can be embedded in process-analytical-technology (PAT) systems, enabling continuous quality assessment of solvents, reagents, catalysts, and process intermediates. ML models capable of handling multivariate spectral backgrounds, complex quenching environments, and varying scattering conditions will provide real-time insight into production environments.

In the long term, the field will converge toward cloud-connected, AI-driven fluorescence infrastructures combining mobile platforms, distributed sensors, adaptive calibration engines, mechanistic ML models, and multimodal fusion. These ecosystems will support seamless sensing pipelines—from spectral acquisition to inference, decision making, and long-term archival—thereby transforming CQD fluorescence sensing into a ubiquitous component of environmental management, personalized health, and industrial automation.

Together, these future pathways point toward a rapidly evolving landscape where CQD fluorescence sensing transcends the laboratory context and becomes a cornerstone of intelligent global monitoring systems.

### 5.4. Standardization framework and community-level development guidelines for CQD-ML fluorescence sensing

To ensure the long-term sustainability, reproducibility, and translational impact of ML-enhanced CQD fluorescence sensing, the establishment of standardized research and reporting frameworks is essential. While current studies demonstrate impressive advancements in sensitivity, classification accuracy, and real-time sensing capabilities, the lack of unified protocols for data acquisition, model evaluation, and material characterization remains a major barrier to field-wide convergence.

At the experimental level, a standardized CQD synthesis and reporting protocol is required to minimize variability in photoluminescence properties across laboratories. Key parameters such as precursor composition, reaction conditions, surface functionalization, quantum yield, particle size distribution, and emission stability should be systematically reported using unified metrics. This would significantly improve dataset consistency and enable reliable cross-study ML model training.

At the data level, the community should move toward the creation of open-access, benchmark fluorescence datasets that include diverse excitation conditions, analyte concentrations, and environmental matrices. Such datasets should be designed to capture real-world variability, including noise characteristics, solvent effects, and instrumental differences, enabling robust evaluation of ML generalization capability. At the computational level, standardized evaluation protocols for ML models are required. Metrics should extend beyond accuracy and include robustness under domain shift, calibration stability, uncertainty quantification, and interpretability of predictions. This is particularly important for CQD-based sensing systems operating in complex environments where model reliability is as critical as predictive performance.

At the system level, cross-platform calibration standards must be established to address variability across optical instruments, excitation sources, and detection systems. Domain adaptation strategies and transfer learning benchmarks should be incorporated into evaluation pipelines to ensure reproducibility across hardware configurations. Finally, a community-driven open framework integrating material synthesis, spectral datasets, ML models, and evaluation protocols would significantly accelerate progress in the field. Such an initiative would enable transparent comparison of methodologies, reduce redundancy in experimental design, and promote reproducible innovation. Overall, the adoption of standardized guidelines and shared infrastructures is a critical step toward transforming CQD fluorescence sensing from isolated laboratory demonstrations into a mature, scalable, and industrially deployable technology.

### 5.5. Bridging photophysical limitations and sensing performance in CQD fluorescence systems

A critical challenge in CQD-based fluorescence sensing lies in the direct linkage between intrinsic photophysical phenomena and measurable analytical performance. Key limitations such as spectral heterogeneity and excitation-dependent emission are not merely material-level characteristics, but fundamental factors that govern sensing reliability, sensitivity, and quantitative accuracy. Spectral heterogeneity leads to broadened and overlapping emission profiles, which reduce analyte distinguishability and degrade selectivity in multicomponent systems. Similarly, excitation-dependent emission introduces variability in spectral signatures under different excitation conditions, resulting in inconsistent calibration curves and reduced reproducibility of LOD estimations.

From a performance perspective, these effects collectively manifest as reduced signal-to-noise ratio, diminished classification accuracy, and unstable quantification across varying environmental and instrumental conditions. In practical sensing scenarios, especially in complex matrices such as biological fluids or environmental samples, these photophysical instabilities significantly amplify analytical uncertainty and hinder reliable deployment. Future ML-driven sensing frameworks are expected to explicitly address these limitations by learning invariant representations of fluorescence signals that are robust to excitation variability and spectral distortion. By mapping heterogeneous and excitation-dependent spectra into stable latent feature spaces, ML can partially decouple photophysical variability from analytical outputs, thereby improving robustness in both single-analyte detection and multi-analyte classification tasks. In this context, ML acts as a compensatory layer that translates complex photophysical behavior into reliable sensing performance metrics. Importantly, establishing this direct bridge between CQD photophysics and sensing performance is essential for designing next-generation analytical systems. It provides a unified framework where material-level behavior, spectral response, and computational modeling are jointly considered to optimize detection limits, selectivity, and operational stability.

## 6. Conclusion

Machine-learning-enabled fluorescence signal processing has emerged as a transformative direction for enhancing the analytical power, stability, and interpretability of CQD-based sensing systems. By addressing the intrinsic photophysical complexities of CQDs—including spectral heterogeneity, excitation-dependent emission, and surface-state dynamics, and environmental sensitivity— ML methods provide a scalable framework capable of extracting reliable information even under low signal-to-noise conditions. Recent advancements illustrate how noise suppression, spectral decomposition, feature extraction, and automated decision-making architectures substantially improve detection accuracy across chemical, biological, and environmental applications.

Beyond performance gains, the convergence of photophysics and data science has begun to shift CQD sensing toward more intelligent and autonomous paradigms. Hybrid physics-informed models promise deeper mechanistic interpretability, generative augmentation offers robust solutions to data scarcity, and mobile or wearable implementations demonstrate the practicality of real-time, user-integrated fluorescence analytics. These developments collectively indicate a broader transition from static, laboratory-dependent workflows to adaptive, distributed, and context-aware sensing infrastructures.

Looking ahead, the field is poised for rapid expansion driven by autonomous closed-loop systems, multimodal architectures unifying temporal and spectral domains, and globally scalable cloud-connected networks. Realizing these opportunities will require interdisciplinary collaboration across materials science, spectroscopy, machine learning, and embedded hardware. As these technologies continue to mature, ML-enhanced CQD fluorescence sensing is expected to evolve into a highly reliable, interpretable, and universally deployable platform for next-generation analytical and diagnostic applications.

## Conflicts of interest

The authors declare that they have no known competing financial interests or personal relationships that could have appeared to influence the work reported in this paper.

## Data Availability

Data sharing not applicable to this article as no datasets were generated or analysed during the current study.

## References

[cit1] Ishak W. N., Lim Y. P., Bakar N. F., Ng L. Y., Tan H. L. (2026). Carbon quantum dots for photocatalytic wastewater treatment: Mechanistic insights and economic feasibility. J. Environ. Manage..

[cit2] Yadav N., Sharma A., Yano T. A., Anthopoulos T. D., Mishra V. (2026). Next-generation functionalized carbon quantum dots: Synthesis approach, structure, photoluminescence mechanism, and multifunctional applications. Coord. Chem. Rev..

[cit3] Liu Z., Yang F., Zhang G., Liao Y., Cui M., Li H., Hu L., Mo H. (2026). Carbon quantum dots: A green and efficient material for fruits and vegetables preservation. Food Rev. Int..

[cit4] Meng L., Ding P., Ou M., Zhang Y. (2026). Houttuynia cordata-DES carbon quantum dots for Cr6+ detection and biological applications. Chem. Biol. Technol. Agric..

[cit5] Fang Y. (2026). Advancements and prospects of carbon quantum dots-based sensors for organophosphorus pesticides detection. Monatsh. Chem..

[cit6] Sen B., Sarma H. (2026). Carbon quantum dots as multifunctional nanomaterials for sustainable optoelectronic biosensing and green photonics. Commun. Mater..

[cit7] Alam M. W., Radhakrishnan K., Saravanan P., Kumar J. V., Ahmad M. M., Sadaf S., Ansari S. A., Nivetha M. S. (2026). Carbon Quantum Dots in Food and Environmental Safety: Advances in Sensing Technologies for Contaminant Detections. J. Food Process. Eng..

[cit8] Eliboev I., Ishankulov A., Berdimurodov E., Chulpanov K., Nazarov M., Jamshid B., Toshpulotov B., Tukhtaeva R., Demir M., Rashidova K., Jalilov F. (2025). Advancing analytical chemistry with carbon quantum dots: a comprehensive review. Anal. Methods.

[cit9] Beshahwored S. S. (2025). Carbon quantum dots (CQDs) in forensic investigations: a review of current applications and future perspectives. RSC Adv..

[cit10] Alqahtani T., Alqahtani A., Alshehri A., Almrasy A. A. (2025). Novel B, N-doped carbon quantum dot fluorescence quenching method for sensitive and green determination of lisinopril: mechanistic insights from quantum calculations and sustainable analytical approach. RSC Adv..

[cit11] Haque S. M., Umar Y., Kabir A. (2025). Advanced Chemometric Techniques for Environmental Pollution Monitoring and Assessment: A Review. Chemosensors.

[cit12] Pratyusha P. L., Venkateswararao A., Tulasi N. L. (2025). A Brief Description Of Chemometric Techniques And Their Types. Eur. J. Biomed. Res..

[cit13] Kaur G., Sharma A., Sharma V. (2025). Rapid and non-destructive FTIR-chemometric method for polybag analysis: distinguishing biodegradable from non-biodegradable materials. Next Res..

[cit14] de Jong F., Martin C., Hofkens J., Van der Auweraer M. (2025). Data Analysis Methods in Time-Resolved Fluorescence Spectroscopy: A Tutorial Review. Chem.–Eur. J..

[cit15] Ferreira D. S., Silva R. A., Pacheco G. M., Pereira-Filho E. R., Pereira F. M. (2026). A review of multivariate modelling for x-ray fluorescence techniques. Chemom. Intell. Lab. Syst..

[cit16] Guembe-Garcia M., Magnaghi L. R., Franceschi G. E., Bova A., Biesuz R. (2026). Multi-Way Data Analysis Nowadays: Taking Advanced Chemometric Tools to Everyday Analytical Chemistry Applications. Chemosensors.

[cit17] Workman Jr J. (2025). Recent Research in Chemometrics and AI for Spectroscopy, Part I: Foundations, Definitions, and the Integration of Artificial Intelligence in Chemometric Analysis. Spectroscopy.

[cit18] Zhao Y., Zhang Z., Hu B., Liu J., Wang X., Zou L., Yu T. (2025). Interpretable data-driven chemometric approach for predicting non-optically active water quality parameters using ultraviolet-visible-near infrared absorption spectroscopy and physical-chemical measurements. Spectrochim. Acta, Part A.

[cit19] Schug D., Kovach T. J., Wolfe M. A., Benson J., Park S., Dodson J. P., Corrigan J., Eriksson M. A., Zwolak J. P. (2025). Automation of quantum dot measurement analysis via explainable machine learning. Mach. Learn.: Sci. Technol..

[cit20] Lee T., Song M., Baek J., Jung Y. N., Choi G. (2026). Advancing quantum-dot optoelectronics: Simulation and machine learning for performance optimization. Chem. Phys. Rev..

[cit21] Babu K. (2025). Luminescent perovskite quantum dots: progress in fabrication, modelling and machine learning approaches for advanced photonic and quantum computing applications. J. Lumin..

[cit22] Taylor J. R., Das Sarma S. (2025). Neural network based deep learning analysis of semiconductor quantum dot qubits for automated control. Phys. Rev. B.

[cit23] Matyszczak G., Krawczyk K., Yedzikhanau A. (2025). Computational modeling of properties of quantum dots and nanostructures: from first principles to artificial intelligence (a review). Nanomaterials.

[cit24] El-Azazy M., Osman A. I., Nasr M., Ibrahim Y., Al-Hashimi N., Al-Saad K., Al-Ghouti M. A., Shibl M. F., Al-Muhtaseb A. A., Rooney D. W., El-Shafie A. S. (2024). The interface of machine learning and carbon quantum dots: From coordinated innovative synthesis to practical application in water control and electrochemistry. Coord. Chem. Rev..

[cit25] Salahinejad M. and Roozbahani A., Applications of Machine Learning Predictive Modeling for Carbon Quantum Dots, In Materials Informatics II: Software Tools and Databases 2025 Mar 15, Springer Nature, Switzerland Cham, pp. , pp. 81–108

[cit26] Malashin I., Martysyuk D., Nelyub V., Borodulin A., Gantimurov A., Tynchenko V. (2026). A review of applications of machine learning in quantum dots research. Discover Nano.

[cit27] Duman A. N., Jalilov A. S. (2024). Machine learning for carbon dot synthesis and applications. Mater. Adv..

[cit28] Chang C. Y., Venkatesan S., Herman A., Wang C. L., Teng H., Lee Y. L. (2023). Carbon quantum dots with high quantum yield prepared by heterogeneous nucleation processes. J. Alloys Compd..

[cit29] Yadav P. K., Chandra S., Kumar V., Kumar D., Hasan S. H. (2023). Carbon quantum dots: synthesis, structure, properties, and catalytic applications for organic synthesis. Catalysts.

[cit30] Guan X., Li Z., Geng X., Lei Z., Karakoti A., Wu T., Kumar P., Yi J., Vinu A. (2023). Emerging trends of carbon-based quantum dots: nanoarchitectonics and applications. Small.

[cit31] Chawre Y., Satnami M. L., Kujur A. B., Ghosh K. K., Nagwanshi R., Karbhal I., Pervez S., Deb M. K. (2023). Forster resonance energy transfer between multicolor emissive N-doped carbon quantum dots and gold nanorods for the detection of H2O2, glucose, glutathione, and acetylcholinesterase. ACS Appl. Nano Mater..

[cit32] Feld L. G., Boehme S. C., Morad V., Sahin Y., Kaul C. J., Dirin D. N., Rainò G., Kovalenko M. V. (2024). Quantifying forster resonance energy transfer from single perovskite quantum dots to organic dyes. ACS Nano.

[cit33] Giordano M. G., Seganti G., Bartoli M., Tagliaferro A. (2023). An overview on carbon quantum dots optical and chemical features. Molecules.

[cit34] Mousa M. A., Abdelrahman H. H., Fahmy M. A., Ebrahim D. G., Moustafa A. H. (2023). Pure and doped carbon quantum dots as fluorescent probes for the detection of phenol compounds and antibiotics in aquariums. Sci. Rep..

[cit35] Nguyen K. G., Huš M., Baragau I. A., Puccinelli E., Bowen J., Heil T., Nicolaev A., Andrews D., Sajjad M. T., Dunn S., Kellici S. (2024). Controlling the optoelectronic properties of nitrogen-doped carbon quantum dots using biomass-derived precursors in a continuous flow system. Carbon.

[cit36] Shabbir H., Csapó E., Wojnicki M. (2023). Carbon quantum dots: the role of surface functional groups and proposed mechanisms for metal ion sensing. Inorganics.

[cit37] Kumar P., Dua S., Kaur R., Kumar M., Bhatt G. (2022). A review on advancements in carbon quantum dots and their application in photovoltaics. RSC Adv..

[cit38] Dua S., Kumar P., Pani B., Kaur A., Khanna M., Bhatt G. (2023). Stability of carbon quantum dots: a critical review. RSC Adv..

[cit39] Sengottuvelu D., Shaik A. K., Mishra S., Ahmad H., Abbaszadeh M., Hammer N. I., Kundu S. (2022). Multicolor nitrogen-doped carbon quantum dots for environment-dependent emission tuning. ACS Omega.

[cit40] Mondal T. K., Kapuria A., Miah M., Saha S. K. (2023). Solubility tuning of alkyl amine functionalized carbon quantum dots for selective detection of nitroexplosive. Carbon.

[cit41] Wei J., Zhang X., Mugo S. M., Zhang Q. (2022). A portable sweat sensor based on carbon quantum dots for multiplex detection of cardiovascular health biomarkers. Anal. Chem..

[cit42] Xu N., Huang H., Ouyang H., Wang H. (2019). Preparation of the heterojunction catalyst N-doping carbon quantum dots/P25 and its visible light photocatalytic activity. Sci. Rep..

[cit43] Thulasinathan B., Sujatha D., Murugan S., Panda S. K., Veerapandian M., Manickam P. (2023). DNA-functionalized carbon quantum dots for electrochemical detection of pyocyanin: a quorum sensing molecule in Pseudomonas aeruginosa. Biosens. Bioelectron..

[cit44] Zhao Y., Song J., Yang Q., Li Y., Liu Z., Yang F. (2024). Real-time visualization of carbon quantum dot transport in homogeneous and heterogeneous porous media. Environ. Sci.: Nano.

[cit45] Tao X., Liao M., Wu F., Jiang Y., Sun J., Shi S. (2022). Designing of biomass-derived carbon quantum dots@ polyvinyl alcohol film with excellent fluorescent performance and pH-responsiveness for intelligent detection. Chem. Eng. J..

[cit46] Deka M. J., Chowdhury D., Nath B. K. (2022). Recent development of modified fluorescent carbon quantum dots-based fluorescence sensors for food quality assessment. Carbon Lett..

[cit47] Patra S., Singh M., Subudhi S., Mandal M., Nayak A. K., Sahu B. B., Mahanandia P. (2023). One-step green synthesis of in–situ functionalized carbon quantum dots from Tagetes patula flowers: applications as a fluorescent probe for detecting Fe3+ ions and as an antifungal agent. J. Photochem. Photobiol., A.

[cit48] Zeng L., Yang S., Chen Q., Fu W., Wu M., Oleszczuk P., Pan B., Xing B. (2024). The critical role of electron donating rate of pyrogenic carbon in mediating the degradation of phenols in the aquatic environment. Water Res..

[cit49] Cheng Y., Ye Y. X., Huang Y., Yan H., Zhang L., Zhu F., Ouyang G. (2024). Outstanding photocatalytic degradation performance under low light intensity via mitigating the quenching reaction of oxygen-centered organic radicals and oxygen. Appl. Catal., B.

[cit50] Kim J. (2024). Recent progresses and challenges in colloidal quantum dot light-emitting diodes: a focus on electron transport layers with metal oxide nanoparticles and organic semiconductors. Nanoscale Horiz..

[cit51] Qureshi Z. A., Dabash H., Ponnamma D., Abbas. M. K. (2024). Carbon dots as versatile nanomaterials in sensing and imaging: Efficiency. Heliyon.

[cit52] Zhang Z., Yang C., Song X., Yu Q., Zhao Z., Zhao H., Zhang Y. (2024). Embedding carbon quantum dots in cell envelops to accelerate electron transfer for microbial advanced oxidation. Chem. Eng. J..

[cit53] Ishak N., Galar P., Mekkat R., Grandcolas M., Soos M. (2025). Fine-tuning photoluminescence and photocatalysis: Exploring the effects of carbon quantum dots synthesis and purification on g-C3N4. Colloids Surf., A.

[cit54] Franco C. R., Miranda-Andrades J. R., Toloza C. A., Larrudé D. G., Maqueira-Espinosa L., Aucelio R. Q., De Falco A., Pedrozo-Peñafiel M. J. (2023). Determination of thiomersal and mercurial residues by photo-degradation and flow injection analysis with luminescence probing using carbon quantum dots prepared from thiourea. Talanta Open.

[cit55] Tyutrin A. A., Wang R., Martynovich E. F. (2022). Luminescent properties of carbon quantum dots synthesized by microplasma method. J. Lumin..

[cit56] Dutta S. D., Moniruzzaman M., Hexiu J., Sarkar S., Ganguly K., Patel D. K., Mondal J., Lee Y. K., Acharya R., Kim J., Lim K. T. (2023). Polyphenolic carbon quantum dots with intrinsic reactive oxygen species amplification for two-photon bioimaging and in vivo tumor therapy. ACS Appl. Mater. Interfaces.

[cit57] Wang Q., Zhang Q., He H., Feng Z., Mao J., Hu X., Wei X., Bi S., Qin G., Wang X., Ge B. (2022). Carbon dot blinking fingerprint uncovers native membrane receptor organizations via deep learning. Anal. Chem..

[cit58] Xiong Y., Huang Q., Canady T. D., Barya P., Liu S., Arogundade O. H., Race C. M., Che C., Wang X., Zhou L., Wang X. (2022). Photonic crystal enhanced fluorescence emission and blinking suppression for single quantum dot digital resolution biosensing. Nat. Commun..

[cit59] Song L., Zhang X., Zhu S., Xu Y., Wang Y. (2022). Hydrogen spillover effect enhanced by carbon quantum dots activated MoS2. Carbon.

[cit60] Saini B., Khamari L., Mukherjee T. K. (2022). Kinetic and mechanistic insight into the surfactant-induced aggregation of gold nanoparticles and their catalytic efficacy: importance of surface restructuring. J. Phys. Chem. B.

[cit61] Wu Y., Chen Z., Guo H., Li J., Yi H., Yu J., He X., He X. (2024). Fluorescence separation based on the spatiotemporal Gaussian mixture model for dynamic fluorescence molecular tomography. J. Opt. Soc. Am. A.

[cit62] An Y., Zhang K., Chai Y., Zhu Z., Liu Q. (2023). Gaussian mixture variational-based transformer domain adaptation fault diagnosis method and its application in bearing fault diagnosis. IEEE Trans. Ind. Inf..

[cit63] Pereira J. M. , Kileel J., Kolda T. G.. Tensor moments of gaussian mixture models: Theory and applications. arXiv, 2022, preprint arXiv: 2202.06930, 10.48550/arXiv.2202.06930

[cit64] Vitale N. H., Hassibi A., Soh H. T., Murmann B., Lee T. H. (2024). Inherent stochasticity, noise and limits of detection in continuous and time-gated fluorescence systems. PLoS One.

[cit65] de Jong F., Martin C., Hofkens J., Van der Auweraer M. (2025). Data Analysis Methods in Time-Resolved Fluorescence Spectroscopy: A Tutorial Review. Chem.–Eur. J..

[cit66] Jameson D. M. , Introduction to Fluorescence, CRC press, 2025

[cit67] Gautheron A., Sdika M., Hébert M., Montcel B. (2023). An explicit estimated baseline model for robust estimation of fluorophores using multiple-wavelength excitation fluorescence spectroscopy. IEEE Trans. Biomed. Eng..

[cit68] Ma J., Zuo M., Wu B., Wang W. (2025). An automated baseline correction method for raman spectra based on piecewise polynomial fitting with adaptive window. J. Raman Spectrosc..

[cit69] Schultz J. D., Parker K. A., Sbaiti B., Beratan D. N. (2025). Using machine learning to map simulated noisy and laser-limited multidimensional spectra to molecular electronic couplings. Digital Discovery.

[cit70] Kistenev Y. V., Skiba V. E., Prischepa V. V., Vrazhnov D. A., Borisov A. V. (2022). Super-resolution reconstruction of noisy gas-mixture absorption spectra using deep learning. J. Quant. Spectrosc. Radiat. Transfer.

[cit71] Larsen J. J., Levy L., Asif M. R. (2022). Removal of powerline noise in geophysical datasets with a scientific machine-learning based approach. IEEE Trans. Geosci. Rem. Sens..

[cit72] Wang B., De Lima T. F., Shastri B. J., Prucnal P. R., Huang C. (2022). Multi-wavelength photonic neuromorphic computing for intra and inter-channel distortion compensations in WDM optical communication systems. IEEE J. Sel. Top. Quantum Electron..

[cit73] Abhaynarasimha K. S., Mani V. V., Sellathurai M. (2024). Mitigating nonlinear distortions of high-powered LEDs for VLC using deep neural networks. Opt. Commun..

[cit74] Chakravartula S. S., Moscetti R., Bedini G., Nardella M., Massantini R. (2022). Use of convolutional neural network (CNN) combined with FT-NIR spectroscopy to predict food adulteration: A case study on coffee. Food Control.

[cit75] Aliferis C. and Simon G., Overfitting, underfitting and general model overconfidence and under-performance pitfalls and best practices in machine learning and AI, Artificial intelligence and machine learning in health care and medical sciences: Best practices and pitfalls, 2024, pp. 477–524 39836821

[cit76] Rajvanshi S. , Kaur G., Dhatwalia A., Arunima S. A. and Bhasin A., Research on problems and solutions of overfitting in machine learning. InInternational Conference on Artificial-Business Analytics, Quantum and Machine Learning, Springer Nature Singapore, 2023, Singapore, pp. 637–651

[cit77] Greenacre M., Groenen P. J., Hastie T., d’Enza A. I., Markos A., Tuzhilina E. (2022). Principal component analysis. Nat. Rev. Methods Primers.

[cit78] Bharadiya J. P. (2023). A tutorial on principal component analysis for dimensionality reduction in machine learning. Int. J. Innov. Res. Sci. Eng. Technol..

[cit79] Grossutti M., D’Amico J., Quintal J., MacFarlane H., Quirk A., Dutcher J. R. (2022). Deep learning and infrared spectroscopy: representation learning with a β-variational autoencoder. J. Phys. Chem. Lett..

[cit80] Guo Y., Jin W., Wang W., Guo Z., He Y. (2022). Unsupervised convolutional variational autoencoder deep embedding clustering for Raman spectra. Anal. Methods.

[cit81] Gonzalez C. M., Horrocks T., Wedge D., Holden E. J., Hackman N., Green T. (2023). Anomaly detection in Fourier transform infrared spectroscopy of geological specimens using variational autoencoders. Ore Geol. Rev..

[cit82] Guo H., Grover K., New E. J. (2026). Machine Learning Assisted Fluorescent Sensor Array for Sensing Applications. Adv. Sens. Res..

[cit83] Souza R. C., Duarte J. C., Goldschmidt R. R., Borges Jr I. (2025). Predicting fluorescence emission wavelengths and quantum yields via machine learning. J. Chem. Inf. Model..

[cit84] Ahmad N., Eid G., El-Toony M. M., Mahmood A. (2025). Harnessing machine learning for the rational design of high-performance fluorescent dyes. Spectrochim. Acta, Part A.

[cit85] Bian Q., Wang X. (2025). Machine Learning-Driven Design of Fluorescent Materials: Principles, Methodologies, and Future Directions. Nanomaterials.

[cit86] Mahato K. D., Kumar U. (2025). Leveraging optimized machine learning regression models for measuring the Stokes shift of fluorescent organic dyes. Theor. Chem. Acc..

[cit87] Tian C., Lee Y., Song Y., Elmasry M. R., Yoon M., Kim D. H., Cho S. Y. (2024). Machine-learning-enhanced fluorescent nanosensor based on carbon quantum dots for heavy metal detection. ACS Appl. Nano Mater..

[cit88] Pang T., Tao X., Zhuang P., Wu M., Li J., Huang H., Sun J., Liu J. (2025). Machine Learning-Assisted Carbon Quantum Dot-Enhanced Fluorescent Probe for the Detection of Zn2+ in Sweat. J. Fluoresc..

[cit89] Dager A., Uchida T., Maekawa T., Tachibana M. (2019). Synthesis and characterization of mono-disperse carbon quantum dots from fennel seeds: photoluminescence analysis using machine learning. Sci. Rep..

[cit90] Liu Y., Chen J., Xu Z., Liu H., Yuan T., Wang X., Wei J., Shi Q. (2022). Detection of multiple metal ions in water with a fluorescence sensor based on carbon quantum dots assisted by stepwise prediction and machine learning. Environ. Chem. Lett..

[cit91] Thonghlueng J., Ngernpimai S., Chuaephon A., Phanchai W., Wiwasuku T., Wanna Y., Wiratchawa K., Intharah T., Thanan R., Sakonsinsiri C., Puangmali T. (2023). Dual-responsive carbon quantum dots for the simultaneous detection of cytosine and 5-methylcytosine interpreted by a machine learning-assisted smartphone. ACS Appl. Mater. Interfaces.

